# Advances and Prospects of Lignin-Derived Hard Carbons for Next-Generation Sodium-Ion Batteries

**DOI:** 10.3390/polym17202801

**Published:** 2025-10-20

**Authors:** Narasimharao Kitchamsetti, Sungwook Mhin

**Affiliations:** 1 National & Local United Engineering Laboratory for Power Batteries, Faculty of Chemistry, Northeast Normal University, Changchun 130024, China; 2 Department of Energy and Materials Engineering, Dongguk University, Seoul 04620, Republic of Korea

**Keywords:** lignin-derived hard carbon, sodium ion batteries, feedstock pretreatment, preparation process tuning, post treatment

## Abstract

Lignin-derived hard carbon (LHC) has emerged as a highly promising anode material for sodium-ion batteries (SIBs), owing to its renewable nature, structural tunability, and notable electrochemical properties. Although considerable advancements have been made in the development of LHCs in recent years, the absence of a comprehensive and critical review continues to impede further innovation in the field. To address this deficiency, the present review begins by examining the intrinsic characteristics of lignin and hard carbon (HC) to elucidate the underlying mechanisms of LHC microstructure formation. It then systematically categorizes the synthesis strategies, structural attributes, and performance influences of various LHCs, focusing particularly on how feedstock characteristics and fabrication parameters dictate final material behavior. Furthermore, optimization methodologies such as feedstock pretreatment, controlled processing, and post-synthesis modifications are explored in detail to provide a practical framework for performance enhancement. Finally, informed recommendations and future research directions are proposed to facilitate the integration of LHCs into next-generation SIB systems. This review aspires to deepen scientific understanding and guide rational design for improved LHC applications in energy storage.

## 1. Introduction

Energy is a fundamental pillar of modern societal development [1]. At present, nonrenewable fossil fuels fulfill over 80% of the world’s energy demands [2]. However, the energy crisis and environmental degradation resulting from fossil fuel combustion have spurred a global pursuit of sustainable and environmentally benign alternatives [3,4]. Among these, renewable energy sources, for instance wind and solar, have garnered significant attention, though their inherent intermittency necessitates the development of reliable energy storage systems to stabilize output [5,6]. Electrochemical energy storage technologies have emerged as strong candidates, offering advantages such as high energy density, efficiency, and operational flexibility [7,8]. Lithium-ion batteries (LIBs) currently hold a dominant position in the energy storage sector, primarily because of their mature technology, long lifespan, and high energy capacity [8,9]. Nonetheless, the growing demand from portable electronics and electric vehicles has led to escalating prices [10]. Moreover, lithium (Li)’s limited reserves and their uneven geographic distribution, along with stalled improvements in energy density, raise concerns about the feasibility of LIBs for large-scale grid storage [11,12]. In light of these limitations, SIBs have emerged as promising alternatives, thanks to sodium (Na)’s abundance and low cost [13,14]. However, a key difference lies in the anode: while LIBs typically use graphite, this material is unsuitable for SIBs due to the larger ionic size of Na and the instability of Na-C compounds.

SIBs utilize a working principle and core components very similar to those of LIBs, which allows researchers and manufacturers to draw upon the technological advances already made for LIBs [15]. Yet, one major point of difference lies in the choice of anode material: graphite, the standard anode in LIBs, does not function effectively in SIBs due to the larger size of Na ions and the instability of Na-C compounds [16,17]. This incompatibility has made the development of suitable anode materials a central challenge for the commercial realization and wide-scale use of SIBs [18,19]. Of the many anode options explored to date, C-based materials, especially HCs, stand out as the most promising. Their appeal stems from their natural abundance, straightforward synthesis, structural versatility, and strong Na storage capacity [20,21]. Stevens et al. were the first to demonstrate the potential of HCs for SIBs in 2020 [22], and since then, remarkable laboratory results have been achieved, with some HCs reaching reversible capacities as high as 546 mAh g^−1^ [23] and initial coulombic efficiencies (ICEs) of up to 95% [24]. Nevertheless, moving from lab-scale advances to commercial production requires that HCs offer not only excellent electrochemical properties but also economical and scalable manufacturing processes [25,26]. Considerable research and development are still required to meet these commercialization benchmarks.

Feedstock selection is a pivotal determinant in the structural characteristics, electrochemical performance, and economic viability of HCs [27,28]. Accordingly, the identification of low-cost, sustainable, and readily available precursors is of paramount importance for the scalable production of high-performance HCs [21]. Currently, anthracite, pitch, synthetic polymers (e.g., phenolic resins), and lignocellulosic biomass represent the primary sources used in HC synthesis [29,30,31]. Table 1 outlines the comparative benefits and limitations of these feedstocks [2,25]. Among them, lignocellulosic biomass emerges as a particularly promising candidate due to its abundance, renewability, and low-cost processing characteristics [32,33]. Notably, lignin, the most plentiful natural phenolic polymer, offers unique advantages, including a high C content (>60%), abundant functional groups, and an inherently crosslinked aromatic framework [34,35]. Global lignin production is estimated at approximately 70 million tons annually, predominantly sourced from the paper and cellulosic biofuel sectors [36,37], thereby ensuring an ample and stable supply chain for HC fabrication. Furthermore, lignin’s high C content translates into superior C yields relative to cellulose during carbonization [38]. The reactive functional groups and structural features of lignin also facilitate its integration with other polymers, enabling tunable structural control over HC products [39,40]. Over the past decade, increasing research attention has focused on the development of LHCs [41,42]. Although a variety of review articles have addressed lignin applications in energy storage and C materials, a focused review examining LHCs specifically for SIBs remains absent in the current literature [43,44].

**Figure 1:**
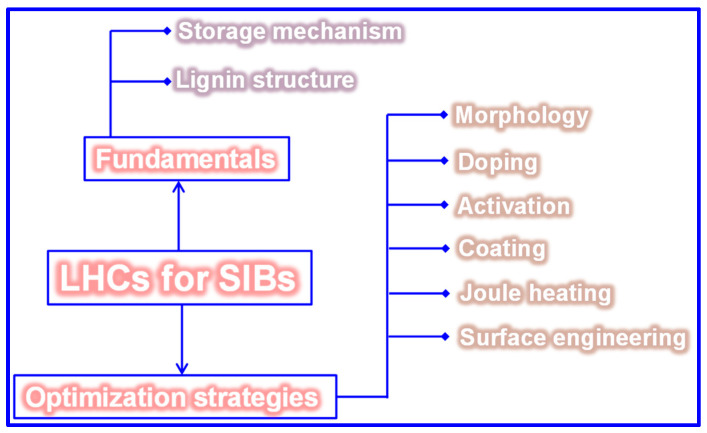
A comprehensive overview of the key topics discussed in the review.

This review presents an in-depth analysis of the fundamentals and recent advancements related to LHCs for SIBs, as depicted in Figure 1. It commences with an overview of lignin’s structural characteristics, classification schemes, and pyrolytic behavior. Next, the fundamental principles of HCs and their Na storage mechanisms are discussed. The article then elaborates on various synthesis methods and the formation pathways of LHCs, summarizing key factors that govern their structural evolution. Special emphasis is placed on optimization strategies, including feedstock pretreatment, process control, and post-synthesis modification techniques. Lastly, prevailing challenges and potential future research directions are critically evaluated. This review aims to offer valuable scientific insights into the structural design of LHCs and to inspire further research toward the effective utilization of lignin.

## 2. Lignin

The structural and physicochemical properties of feedstocks are intrinsically connected to the final architecture of HCs [21,25,45]. Accordingly, a thorough understanding of lignin’s fundamental characteristics is essential, as it informs both the formation pathways of LHCs and the design of effective optimization strategies. In this context, the opening section of this review delivers a succinct overview of lignin’s structural features, classification systems, and pyrolytic behavior, thereby providing a solid basis for the discussions that follow.

### 2.1. Classification and Structure

**Figure 2:**
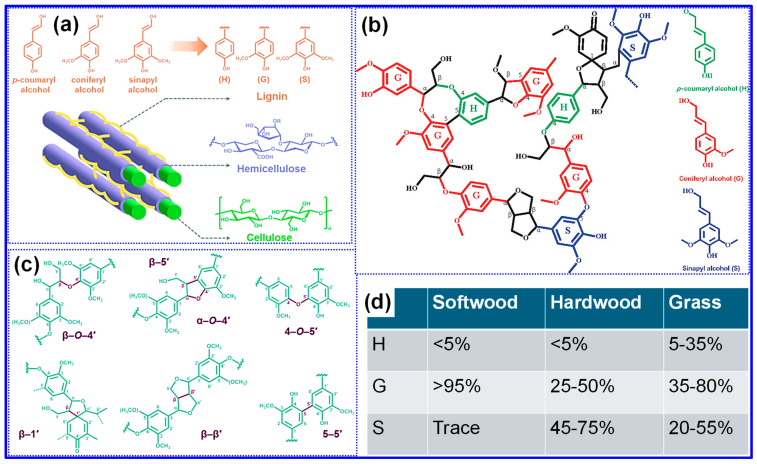
Key aspects of lignin’s structure and chemistry: (**a**) The spatial distribution and core monolignol monomer units. Adapted from [40]. Copyright 2022, Royal Society of Chemistry. (**b**) A schematic representation of the lignin macromolecule. Adapted from [48]. Copyright 2021, Elsevier B.V. (**c**) Common inter-unit linkages present in lignin polymers. Adapted from [40]. Copyright 2022, Royal Society of Chemistry. (**d**) Variations in monolignol composition depending on lignin source. Adapted from [38]. Copyright 2022, Royal Society of Chemistry.

Lignin, one of the three principal components of lignocellulosic biomass [46], is intimately associated with cellulose and hemicellulose through a network of hydrogen bonds (Figure 2a) [40]. Within plant cell walls, lignin plays vital roles in providing mechanical strength and rigidity, protecting against microbial degradation, and facilitating water transport [38]. The lignin content within biomass varies depending on species, tissue type, and maturity, typically ranging from 15 to 39% [36,38]. Following cellulose, lignin ranks as the second most abundant natural polymer globally [46], and due to its high C content, it contributes approximately 30% of the total organic carbon (TOC) on Earth [47].

Lignin does not possess a defined primary structure, highlighting its inherently complex and irregular architecture [49]. Its defining characteristic is its composition of three main monolignol precursors, *p*-coumaryl, coniferyl, and sinapyl alcohol, which give rise to *p*-hydroxyphenyl (H), guaiacyl (G), and syringyl (S) units, respectively (Figure 2a) [40]. These structural units are interconnected through various ester and C-C bonds, producing a heterogeneous, amorphous 3D framework (Figure 2b) [48]. Representative interunit linkages include β-O-4, β-5, α-O-4, 4-O-5, β-1, β-β, and 5-5, as illustrated in Figure 2c [40]. Among these, the β-O-4 bond is particularly noteworthy, accounting for approximately 50% of total linkages and exhibiting relative lability during lignin extraction [50]. Consequently, the β-O-4 content serves as an important metric for evaluating lignin’s applicability. Similarly to its abundance, lignin’s structural features vary with botanical origin [36,38]: softwoods predominantly contain G units, hardwoods comprise both G and S units, and grasses incorporate all three monolignol-derived units (Figure 2d) [38]. Additional structural parameters, such as interunit bonding patterns, oxygenated functional groups, and molecular weight (MW), also differ by source [51]. Empirical studies indicate that hardwood lignin generally has a higher proportion of β-O-4 linkages and phenolic hydroxyl groups but a lower MW compared to softwood lignin [50]. Beyond species, extraction techniques exert significant influence on lignin’s structural characteristics [52], a topic explored in the subsequent section.

Lignin can be categorized according to various criteria; however, the classifications most commonly recognized are based on botanical origin and extraction technique, both of which profoundly affect its structural characteristics [36,38]. Different extraction processes yield distinct lignin types, including kraft lignin, lignosulfonate (sulfite), soda lignin, organosolv lignin, steam-explosion lignin, and enzymatic lignin, among others [38,41]. A comprehensive comparison of their structures and properties is presented in Table 2. The availability of specific lignin types significantly impacts their suitability for commercial HC production. In this regard, lignins sourced from the pulping industry such as kraft, lignosulfonate, and soda lignin, are considered particularly promising. Another crucial factor is feedstock purity, which directly influences the structural integrity and electrochemical performance of HCs [53]. Industrial lignins, especially those derived from papermaking and biofuel production, frequently contain impurities and thus necessitate additional purification prior to application in HC synthesis [38]. Furthermore, MW and polydispersity substantially affect the tunability of the resulting HC structures [36]. For instance, lignosulfonate, characterized by a broad MW distribution and high polydispersity index, poses challenges for precise structural control in LHCs, whereas soda and enzymatic lignins offer greater molecular uniformity. Solubility is also a critical parameter, influencing the feasibility of lignin modification and post-treatment. Notably, lignosulfonate’s high water solubility across a broad pH range has attracted considerable attention for this purpose.

### 2.2. Pyrolysis Behaviors

Pyrolysis represents a critical step in transforming lignin into C-based materials and has been the subject of extensive research [41,51]. The behavior of lignin during pyrolysis is influenced by a range of parameters, including annealing temperature, lignin source, heating rate, pyrolysis atmosphere, and the presence of catalysts [55,56]. This section primarily examines the general pyrolytic characteristics of lignin with a specific emphasis on the influence of annealing temperature.

**Figure 3:**
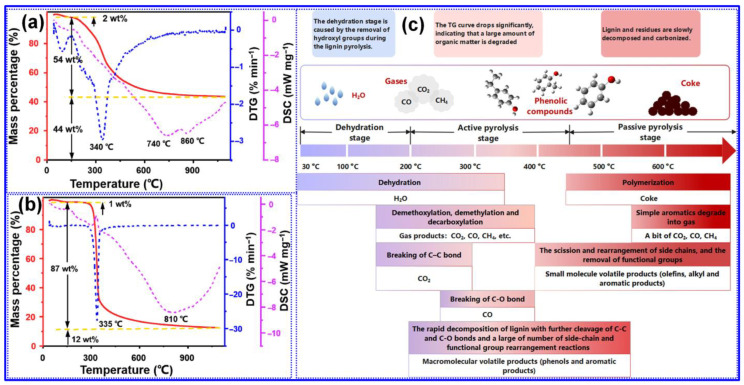
TGA, DTG and DSC plots of (**a**) milled wood lignin and (**b**) microcrystalline cellulose. Adapted from [58]. Copyright 2022, Elsevier B.V. (**c**) Overview of fundamental pyrolysis behavior of lignin. Adapted from [55]. Copyright 2022, Elsevier B.V.

During the pyrolysis of organic precursors, a series of complex chemical transformations take place, including dehydrogenation, hydrogen transfer, removal of O-containing functional groups, molecular rearrangement, and condensation reactions [57]. As depicted in Figure 3a,b, TGA of milled wood lignin and microcrystalline cellulose indicates that lignin pyrolysis is significantly more complex than that of cellulose, owing to lignin’s inherent structural heterogeneity, which results in decomposition over a wide temperature range (150–700 °C) [58]. Lignin pyrolysis generates diverse products distributed among gas, liquid, and solid phases [51]. According to Leng’s group [55], lignin pyrolysis can be divided into three distinct stages based on temperature: dehydration, active pyrolysis, and passive pyrolysis (Figure 3c). Below 200 °C, dehydration predominates, accompanied by minor demethoxylation and decarboxylation reactions. Between 200 and 450 °C, the active pyrolysis phase involves extensive bond cleavage and recombination, leading to substantial structural breakdown and weight loss. Beyond 450 °C, during the passive pyrolysis stage, repolymerization of intermediates into coke occurs, along with further removal of oxygenated functional groups and limited secondary degradation.

In addition to the pyrolytic reactions described above, numerous researchers have advanced detailed mechanisms and insights into lignin carbonization. Studies by Liu’s and Pandey’s groups suggest that lignin degradation initiates with the cleavage of weaker bonds, specifically hydrogen bonds and C-O linkages, with the scission of β-O-4 bonds serving as the primary trigger for chain depolymerization [51]. Xie and colleagues further underscored the pivotal role of free radical intermediates, particularly those arising from β-O-4 bond cleavage, in facilitating the pyrolysis pathway [8]. A more comprehensive mechanistic investigation by Long and coworkers [59] revealed that lignin carbonization proceeds through in situ decomposition coupled with proximal recombination, while also maintaining that the C atoms largely retain their original electronic configurations and elemental composition throughout the process. Recent results from Wang’s group demonstrated that C crystallites derived from lignin exhibit isotropic arrangements during pyrolysis, contrasting with the anisotropic crystallite alignment observed in cellulose-derived C’s [60]. Moreover, these C crystalline domains have been shown to play a crucial role in guiding the development of graphitic layers in HC materials [61].

Overall, lignin holds considerable potential as a precursor for HCs owing to its high C content, inherently aromatic framework, abundance of reactive functional groups, and widespread global availability. However, several intrinsic limitations, including its structural complexity, natural heterogeneity, variability in physicochemical properties, elevated ash content, and the still insufficiently elucidated pyrolysis pathways, present significant challenges to its effective deployment [8]. Consequently, overcoming these constraints has become a central research priority in the advancement of lignin-to-HC technologies.

## 3. HCs

HCs have emerged as among the most promising anodes for SIBs, garnering significant research interest in recent years [62]. With ongoing advancements, an increasingly detailed understanding of HC structures, their structure–performance correlations, and Na storage mechanisms is being established. This section presents a succinct overview of the fundamental characteristics and Na storage principles of HCs, providing a foundation for the subsequent in-depth discussion of LHCs.

### 3.1. Fundamentals

In the field of battery electrode materials, HC is defined as a carbonaceous substance that resists graphitization even at elevated temperatures up to 3000 °C [7]. This field-specific definition is important because the term “hard carbon” originally referred solely to its mechanical hardness in contrast to “soft carbon” [57]. Notably, materials with high mechanical hardness may still be graphitizable [57]. The structural characteristics that prevent graphitization in HCs were first elucidated by Rosalind E. Franklin, who examined carbons of various origins treated between 1000 and 3000 °C [63]. HCs are typically synthesized via pyrolysis of O-rich, crosslinked organic precursors, such as biomass and phenolic resins, at temperatures exceeding 1000 °C [4,10]. Alternatively, inorganic feedstocks with low O-content, including coal and pitch, can also be converted into HCs through a pre-oxidation step prior to high-temperature treatment [64,65].

**Figure 4:**
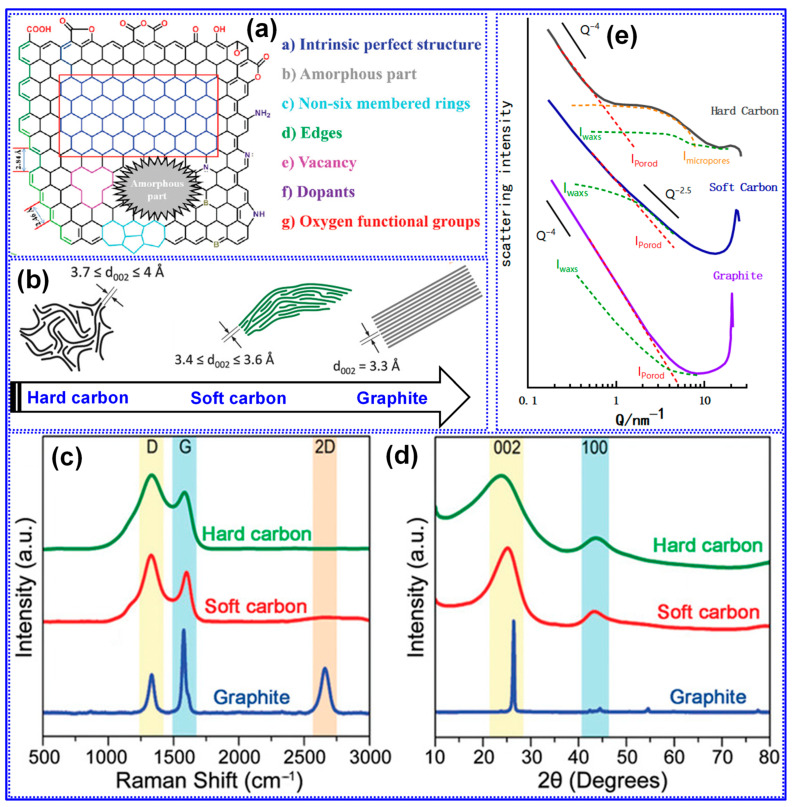
(**a**) The composition of HC microstructure. Adapted from [25]. Copyright 2024, Royal Society of Chemistry. (**b**) A visual glimpse into the intricate microstructures of three distinct C forms. Adapted from [66]. Copyright 2018, Wiley VCH. (**c**) Raman and (**d**) XRD. Adapted from [5]. Copyright 2021, Wiley VCH. (**e**) SAXS spectra of three distinct C forms. Adapted from [68]. Copyright 2022, MDPI.

The microstructure of HC is inherently complex, consisting of a heterogeneous combination of graphitized domains and amorphous regions, which host various structural defects such as vacancies, non-six-membered C rings, heteroatoms, and edge sites (Figure 4a) [25]. In comparison to graphite and soft C, HC is characterized by randomly oriented, curved graphene layers, an expanded interlayer spacing (0.37 < d_002_ < 0.4 nm), and the presence of distinct closed pores (Figure 4b) [66]. These features have been substantiated through a range of characterization techniques, including Raman spectroscopy, XRD, and small-angle X-ray scattering (SAXS) [5]. For instance, as depicted in Figure 4c, the absence of the 2D band (~2685 cm^−1^) in HC’s Raman spectrum specifies the existence of few-layer graphene crystallites (<5 layers). Furthermore, the pronounced D band (~1350 cm^−1^) and the elevated D/G intensity ratio signify a high concentration of defects. The XRD pattern demonstrates a clear blue shift of the (002) peak relative to graphite and soft C, confirming the larger interlayer spacing (Figure 4d). SAXS data further support the presence of microporosity in HC, notably evidenced by a characteristic shoulder peak within the Q range of 1–10 nm^−1^ (Figure 4e) [67,68]. SAXS scattering intensity comprises three components: the macroscopic surface contribution (I_porod_), the internal structural scattering (I_waxs_), and the signal from micropores (I_microporous_) [67]. The detection of closed pores is implied when N_2_ adsorption analysis indicates negligible porosity, yet a shoulder peak appears in the SAXS profile, accompanied by low surface area (SSA) and true density. The intensity of this feature correlates directly with the closed pore volume (P_v_). By contrast, graphite and soft C do not display this SAXS shoulder, indicating the absence of microporosity [68].

### 3.2. Na Storage Mechanisms

**Figure 5:**
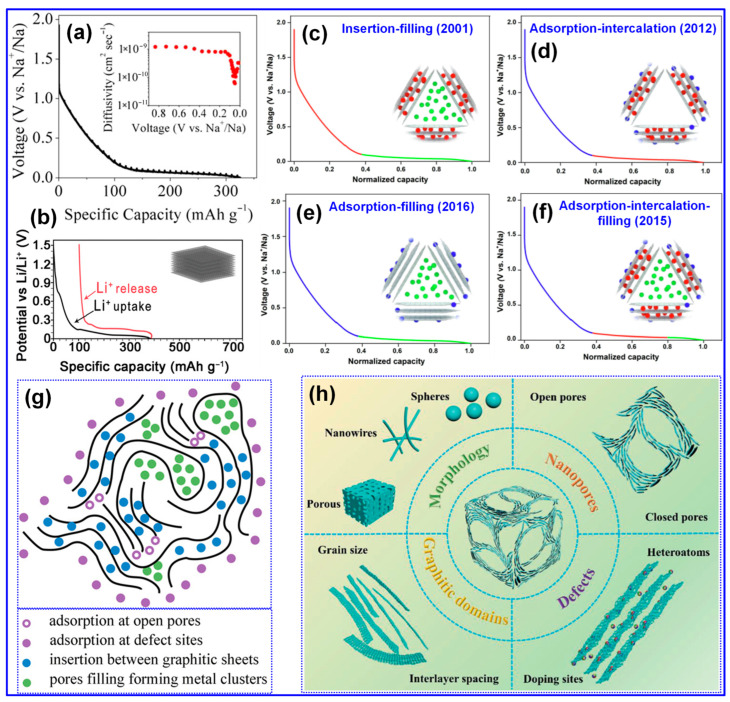
(**a**) Representative voltage–charge profiles of a graphite-based Li half-cell. Adapted from [71]. Copyright 2015, American Chemical Society. (**b**) HC-based Na half-cell. Adapted from [5]. Copyright 2021, Wiley VCH. (**c**–**f**) Four representative models describing Na storage mechanisms. Adapted from [17]. Copyright 2022, Elsevier B.V. (**g**) A schematic illustration of HC microstructure and its principal active sites responsible for Na ion uptake. Adapted from [75]. Copyright 2022, Wiley VCH. (**h**) Schematic depiction of principal structural characteristics of HCs in SIBs. Adapted from [7]. Copyright 2021, Wiley VCH.

A comprehensive understanding of the Na storage mechanism is imperative for the rational design of HCs with improved energy densities [20,69]. Nonetheless, the inherently disordered structure of HCs has rendered the exact Na storage pathway ambiguous and contested, thereby hindering the optimization of HC-based anodes [68]. In contrast to the straightforward Li intercalation in graphite, Na storage within HCs manifests a more intricate behavior [70]. As depicted in Figure 5a,b, the voltage profiles of Li in graphite and Na in HCs diverge significantly; HCs display a gradual slope above 0.1 V and a distinct plateau beneath this threshold [5,71]. To interpret these phenomena, multiple conceptual models have emerged since Stevens et al. proposed the seminal “house-of-cards” and “insertion–filling” paradigms [72]. Evolving perspectives have introduced the “adsorption–insertion,” “adsorption–filling,” and the composite “adsorption–insertion–filling” models (Figure 5c–f) [17]. Recent advances, enabled by sophisticated characterization techniques, have further refined this understanding into hybrid constructs such as the “four-stage” [73] and “extended adsorption–insertion” models [74]. Despite their differences, these frameworks consistently highlight three principal Na storage mechanisms, surface adsorption, interlayer insertion, and nanopore filling, each fundamentally dictated by the microstructural attributes of HCs [75].

The “insertion–filling” mechanism, first introduced by Stevens’s group in 2001 [72], attributed the sloping region of the voltage profile in HCs to the intercalation of Na^+^ ions into graphite-like microcrystal interlayers, while the plateau region was ascribed to Na^+^ accumulation in nanopores (Figure 5c–f). This model was later substantiated by Aniskevich and co-workers through in situ Raman spectroscopy and EIS [76]. Specifically, they observed a reversible G-band shift and changes in charge-transfer resistance in the sloping region, indicating intercalation, while low-frequency Raman spectra confirmed pore-filling in the plateau region. In 2012, Cao’s group [77] proposed the “adsorption–insertion” mechanism, wherein Na^+^ storage was divided into surface/defect site adsorption at high potentials and interlayer insertion at low potentials, with an interlayer spacing of at least 0.37 nm identified as the threshold for Na^+^ intercalation. This interpretation was later supported by Jin’s group [78], who emphasized that enlarging interlayer spacing beyond 0.364 nm could enhance both plateau and overall capacity. Building upon these models, Bommier and colleagues [71] introduced the “adsorption–insertion–filling” mechanism in 2015, describing a stepwise Na^+^ storage process comprising surface adsorption, interlayer insertion, and eventual nanopore filling at voltages below 0.1 V. This tripartite model was validated through in situ XRD, GITT, and ex situ XPS by Zhou [79] and Song’s group [80]. However, in 2016, Zhang et al. [81] and Li et al. [82] challenged this model based on the absence of a (002) XRD peak shift or any significant structural changes upon full sodiation of cotton-derived HCs. They instead proposed an “adsorption–filling” mechanism. Liu et al. [83] further corroborated this alternative interpretation by detecting quasi-metallic Na clusters via in situ XRD during cycling.

As illustrated in Figure 5g, Na ion adsorption predominantly occurs at structural defects, such as non-hexagonal C rings, vacancies, edges, heteroatom sites, as well as at open pores. In contrast, insertion takes place between graphitic C layers, while pore filling is confined to closed micropores [75]. Accordingly, key structural attributes of HCs in SIBs include defects, microporosity, and graphitic microcrystallites, as depicted in Figure 5h [7]. Furthermore, morphological characteristics play a critical role in influencing electrolyte diffusion pathways and Na-ion transport kinetics, thereby directly affecting the rate capability of HCs [7,33].

The primary electrochemical parameters characterizing HCs include specific capacity, ICE, rate capability, and excellent cycling performance [84]. Enhancing Na storage via adsorption, intercalation, and pore filling can be achieved by increasing defect density, introducing more micropores, and expanding the interlayer spacing, strategies that typically boost specific capacity [85]. However, excessive defects and open pores tend to reduce ICE and compromise cycling stability [79,86]. Likewise, while a higher proportion of closed pores favors the pore-filling process and increases capacity, it can also impair rate performance due to slower Na-ion transport [14,34].

The advancement and real-world deployment of HCs in SIBs remain constrained by their inherently complex microstructures, unresolved Na^+^ storage mechanisms, and performance compromises arising from structural heterogeneity. Consequently, the realization of HCs with optimized and well-balanced electrochemical properties requires a thorough and systematic understanding of the fundamental Na^+^ storage processes, as well as the intricate relationships between structural attributes and electrochemical behavior.

## 4. LHCs

It is well recognized that the characteristics of precursor materials play a critical role in determining the structural evolution of HCs [21,45]. Consequently, LHCs display unique structural and electrochemical characteristics when compared to HCs derived from other biomass sources, owing to the distinct physicochemical nature of lignin. This section delves into the synthesis approaches and fundamental characteristics of LHCs, while also providing a comprehensive evaluation of the key factors that influence their structure and electrochemical behavior.

### 4.1. Preparation Techniques and Characteristics

**Figure 6:**
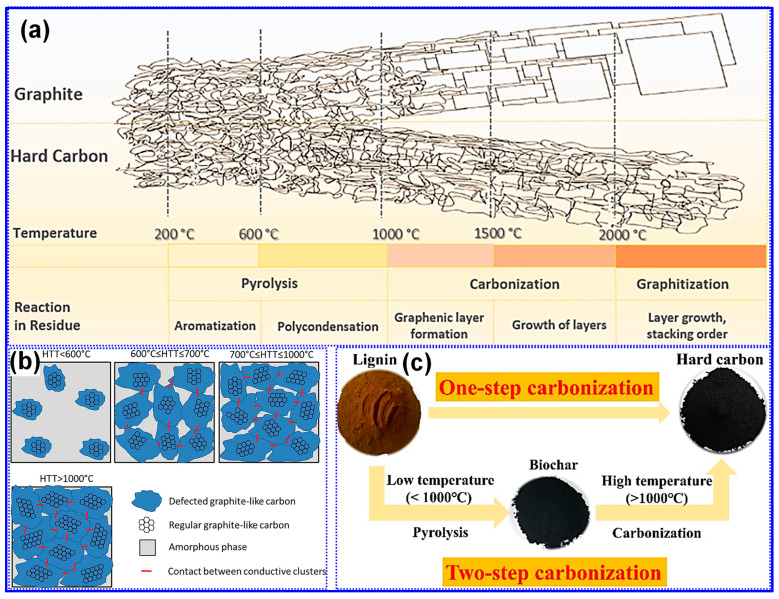
(**a**) Influence of temperature on the carbonization process. Adapted from [57]. Copyright 2019, Elsevier B.V. (**b**) Schematic illustration depicting the evolution of electrical conductivity in lignin-derived C during thermal processing. Adapted from [88]. Copyright 2018, Elsevier B.V. (**c**) Single- and two-step carbonization approaches for the synthesis of HC. Adapted from [91]. Copyright 2025, Elsevier B.V.

Thermal treatment is widely recognized as the primary approach for synthesizing LHCs [87]. As outlined by Dou’s group [57], this process can be categorized into three temperature-dependent stages, pyrolysis (<1000 °C), carbonization (1000–2000 °C), and graphitization (>2000 °C), as illustrated in Figure 6a. During the pyrolysis stage, aromatization and polycondensation reactions give rise to amorphous C structures containing pores and nascent graphite microcrystals. Shao and coworkers [88] analyzed the evolution of electrical conductivity throughout pyrolysis and identified 600 °C as a key transition point (Figure 6b). Below this temperature, graphite microcrystals remain disconnected and exhibit limited conductivity; above it, microcrystal growth and interconnection significantly enhance conductivity [88]. Carbonization initiates beyond 1000 °C, during which graphitic domains mature and open pores collapse, forming closed-pore structures. At temperatures above 2000 °C, graphitic domains expand excessively, resulting in C materials with reduced suitability for SIBs. As such, the optimal thermal treatment range for HC production typically falls between 1000 and 1600 °C, aligning with the carbonization stage [89,90].

Lignin’s hyperbranched architecture, high O-content, and intricate molecular network make it particularly amenable to direct carbonization for HCs synthesis [87]. Two predominant carbonization strategies are employed: one-step and two-step methods (Figure 6c) [91]. The one-step approach involves direct heating of lignin to the desired carbonization temperature, while the two-step method incorporates an initial low-temperature pyrolysis to generate biochar, followed by high-temperature treatment for carbonization [17,92]. Although the one-step method is operationally straightforward, comparative studies have demonstrated that the two-step approach yields HCs with improved electrochemical properties [93]. Li’s group [92] reported that preliminary pyrolysis promotes interlayer expansion, reduces open porosity, and enhances the formation of O-containing functional groups. Similarly, Alvin and colleagues [93] observed partial graphitization during the pyrolysis stage and noted that the one-step route may restrict structural reconfiguration due to limited thermal processing time, thereby producing less favorable C microstructures. In contrast, the two-step method allows for more extensive atomic rearrangement, facilitating the growth of larger graphitic domains, increased interlayer spacing, and the formation of closed pores, all contributing to enhanced Na storage capability in HCs [94].

**Figure 7:**
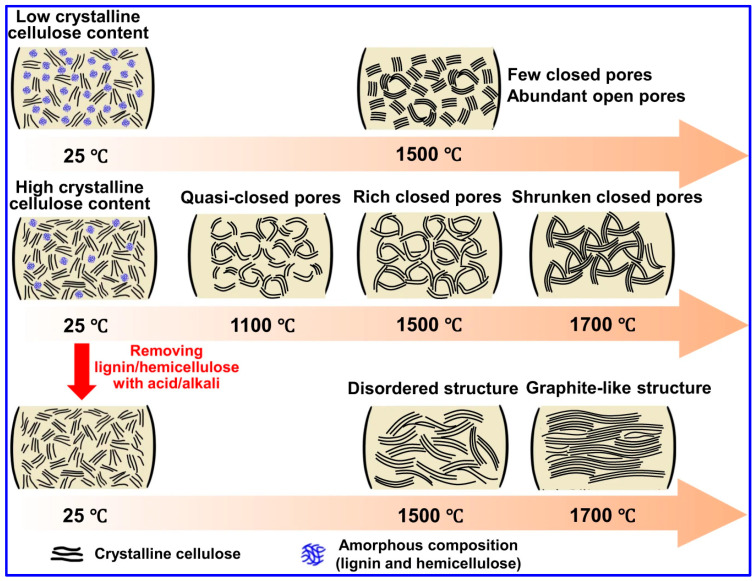
Investigating the contributions of lignin, cellulose, and hemicellulose to the development of closed-pore structures. Adapted from [45]. Copyright 2023, Springer Nature.

Recent investigations by Long and collaborators [59] have classified LHCs as part of the sp^2^-sp^3^ hybrid C family, characterized by sp^2^-bonded graphene domains interconnected by sp^3^ C atoms. Tang and coworkers [45] explored the individual roles of lignin, cellulose, and hemicellulose in the formation of closed pores during carbonization. Their results indicate that crystalline cellulose forms extended graphene layers, which function as pore walls, whereas lignin and hemicellulose inhibit excessive graphitic growth and facilitate sheet contraction, ultimately promoting pore closure (Figure 7). Corroborating these findings, Huang’s team [95] selectively removed lignin and hemicellulose from bamboo fibers using NaOH, thereby enriching cellulose content. This modification improved structural order and enhanced the transition of open to closed pores during high-temperature treatment. Similarly, Shang’s group [96] observed that lower lignin content is conducive to closed-pore formation. Additional studies have confirmed that LHCs generally exhibit a nonporous morphology, which is attributed to the aromatic H, G, and S units present in lignin. In contrast, HCs derived from cellulose and hemicellulose tend to be porous due to their abundant hydroxyl groups [37,97].

In addition, Lotfabad’s group [98] observed that LHCs typically possess smaller graphitic domain sizes than those derived from cellulose, suggesting cellulose’s greater propensity for forming extended graphitic structures. Complementary findings by Wu and colleagues [58] revealed that under identical carbonization temperatures, LHCs exhibited lower sp^2^ C content and a higher concentration of defects relative to cellulose-derived HCs. Similar observations were reported by Feng’s group [99], who removed cellulose and hemicellulose from lignocellulosic biomass prior to carbonization, further supporting these trends. Nonetheless, some studies present conflicting results [60,96]. Shang and collaborators [96] proposed that lignin decomposition during pyrolysis and carbonization may facilitate the formation of large crystalline domains, potentially disrupting the random stacking of graphene layers. In a comparative study, Song’s team [100] analyzed the microstructural evolution of HCs derived from lignin and starch. They concluded that lignin-based HCs exhibited fewer structural defects, larger interlayer spacings, and fewer closed pores, whereas starch-derived HCs showed the opposite trends: greater defect density, reduced interlayer spacing, and more closed pores.

**Figure 8:**
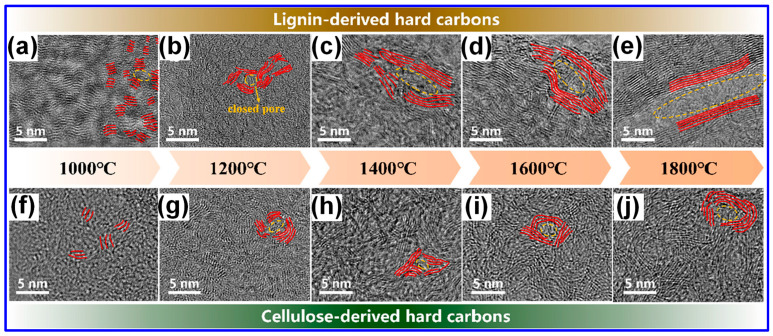
The evolution of C microcrystalline structures in lignin and cellulose with increasing temperature. Adapted from [60]. Copyright 2024, American Chemical Society.

Our study indicates that lignin undergoes transformation into microcrystalline C structures more readily than cellulose during both carbonization and graphitization stages [60,61]. These observations imply that the C microcrystalline seeds formed at lower temperatures from lignin and cellulose differ in spatial arrangement, which in turn influences the resulting graphitic domain architectures in LHCs and cellulose-derived HCs. As illustrated in Figure 8, lignin-derived seeds exhibit an isotropic configuration that promotes the development of long-range, ordered graphite-like domains under high-temperature treatment. In contrast, cellulose-derived seeds form anisotropic structures, yielding smaller, less ordered graphene-like domains with random stacking [60]. It is well recognized that both lignin’s inherent characteristics and the specifics of its thermal processing are pivotal in shaping the structure of the resulting HCs [5]. Accordingly, the presence of seemingly contradictory findings in the literature is justifiable. Although this structural complexity complicates the definitive characterization of LHCs, it simultaneously provides valuable opportunities for targeted microstructural engineering.

**Figure 9:**
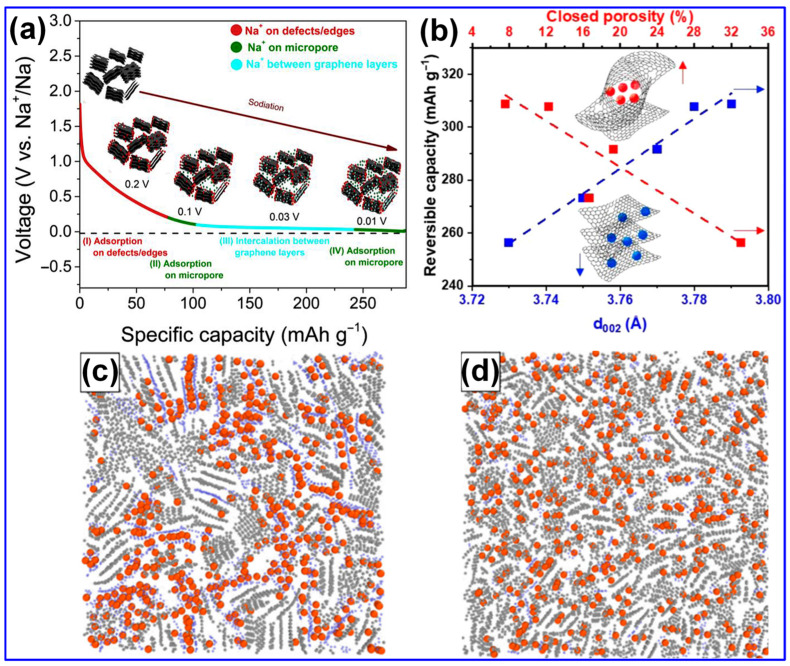
Na storage processes in LHCs. (**a**) Schematic representation of Na-ion storage pathways in LHCs. Adapted from [42]. Copyright 2019, Elsevier B.V. (**b**) Correlation of LHCs’ reversible capacity with closed porosity and interlayer spacing. Adapted from [101]. Copyright 2023, American Chemical Society. 1 ns simulation snapshots showing Na ion distribution (red) in LHCs: crystalline C (gray) and amorphous C (blue). Na storage in the (**c**) amorphous and (**d**) crystalline domains. Adapted from [102]. Copyright 2021, American Chemical Society.

Recent investigations have provided new insights into the Na storage mechanisms within LHC materials. Alvin’s team [42] proposed a comprehensive, multi-stage model that links specific structural features of LHCs to their sodiation behavior across both sloping and plateau voltage regions. This model comprises four distinct stages: (1) initial Na adsorption at defect sites; (2) subsequent adsorption in micropores, occurring primarily within the sloping voltage region (>0.1 V); (3) intercalation of Na ions between graphene layers, corresponding to the plateau region (<0.1 V); and (4) final pore filling within closed pores near the cutoff potential (Figure 9a). Alternatively, Escamilla-Perez and colleagues [101] proposed a different mechanistic pathway based on correlations between reversible capacity and key structural parameters, including interlayer spacing and closed porosity. Their findings indicate that larger interlayer spacing enhances capacity, while increased closed porosity has the opposite effect, thereby favoring an “adsorption–insertion” mechanism (Figure 9b). Complementary insights from Kizzire’s group [102], using reactive molecular dynamics (MD) simulations, demonstrate that Na ions preferentially adsorb on curved graphene surfaces. Moreover, moderate surface curvature and the presence of smaller crystalline domains were found to facilitate more rapid Na diffusion (Figure 9c,d).

### 4.2. Factors Influencing the Performance

The structural and electrochemical characteristics of HCs are widely recognized to be closely dependent on both the nature of the precursor materials and the conditions under which they are synthesized [103]. A deep understanding of these correlations is critical for the rational design of HCs with tailored properties. In this section, we systematically review the principal parameters that govern HC structure and performance, with an emphasis on graphitic domain formation, defect density, P_v_, SSA, and Na storage-related metrics, including specific capacity, ICE, and long-term cycling performance (Table 3).

#### 4.2.1. Feedstock

**Figure 10:**
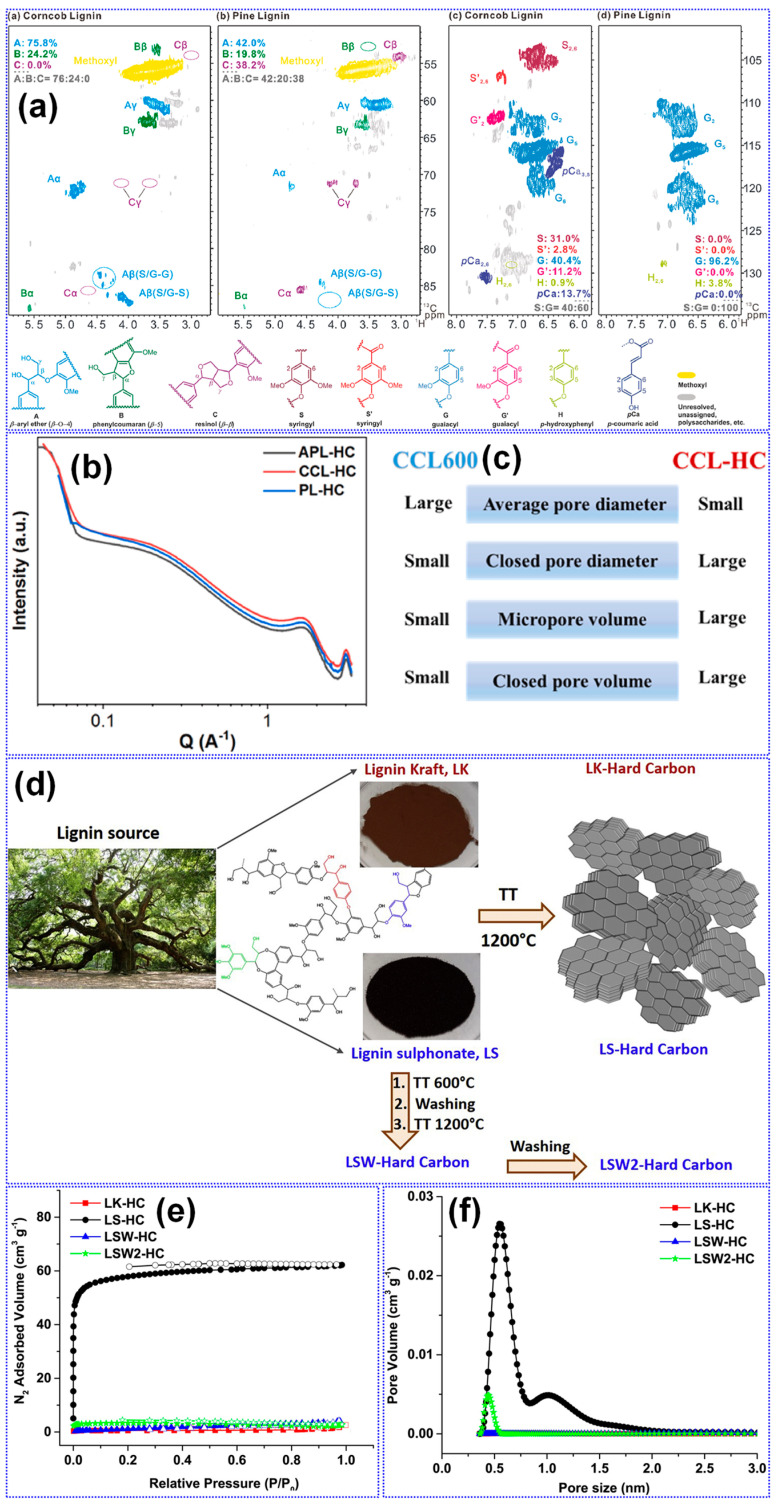
Impact of lignin source on HC structure. (**a**) Partial 2D NMR spectra highlighting key structural features of lignin and (**b**) SAXS profiles and (**c**) pore characteristics of LHCs. Adapted from [116]. Copyright 2023, Elsevier B.V. Impact of extraction methods on HC structure. (**d**) Schematic illustration of lignin-to-HC conversion process. (**e**) N_2_ adsorption–desorption isotherms and (**f**) pore size distribution of LHCs. Adapted from [89]. Copyright 2019, Elsevier B.V.

The structural characteristics of lignin are primarily influenced by its botanical origin and extraction technique [38]. Meng’s team [116] investigated the impact of lignin source on the microstructure and Na storage performance of HCs derived from corn cob, pine, and acetylated pine lignins, all carbonized at 1300 °C. Corn cob lignin, characterized by a high content of β-O-4 and β-5 linkages and a composition of G, H, and S units, yielded HCs with the highest closed P_v_ and plateau capacity. This was attributed to the transformation of micropores into closed pores at elevated temperatures (Figure 10a–c). In contrast, pine-derived lignins, enriched in β-β linkages and G units, generated macropores that inhibited closed pore formation (Table 3). Ghimbeu and colleagues [89] compared kraft and sulfonated lignins carbonized at 1200 °C, finding that kraft-based HCs had lower SSA and P_v_ but higher ICE due to reduced ash content (Figure 10d–f). This was further confirmed by washing sulfonated LHCs, which reduced SSA from 180 to 5.6 m^2^ g^−1^. Nonetheless, the limited structural characterization of lignin feedstocks presents a significant challenge in establishing definitive structure–property correlations.

**Figure 11:**
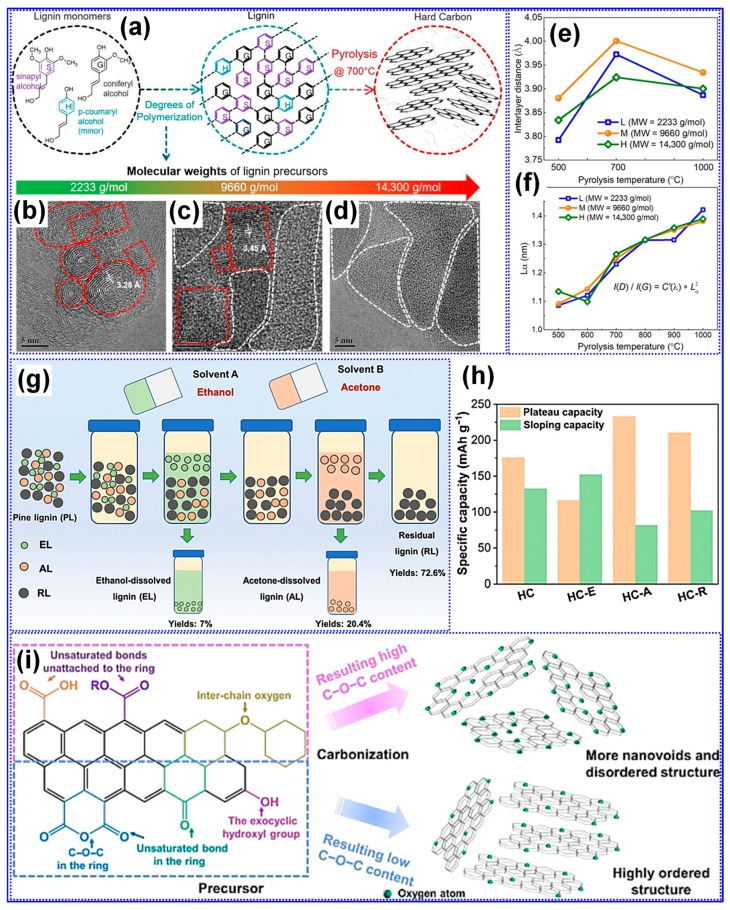
Impact of lignin MW on HC structure. (**a**–**d**) Schematic of LHC fabrication and corresponding TEM micrographs. (**e**,**f**) Interlayer spacing and nanocrystalline size of LHCs. Adapted from [104]. Copyright 2022, Elsevier B.V. Impact of lignin fractionation on LHC structure and performance. (**g**) Schematic diagram showing the steps involved in lignin fractionation. (**h**) The sloping and plateau capacities of LHCs. Adapted from [105]. Copyright 2024, Royal Society of Chemistry. (**i**) Impact of feedstock O-functionalities on HC microstructure. Adapted from [118]. Copyright 2023, Springer Nature.

MW is a critical physical parameter that significantly influences lignin carbonization behavior and the resulting structure of LHCs [117]. Wu’s group [104] conducted a systematic investigation into how varying lignin MWs affect HC structure across different carbonization temperatures. Their study revealed that while parameters such as graphitic microcrystal size (L_a_) and defect density (I*_D_*/I*_G_*) were primarily dependent on thermal treatment, porosity exhibited a strong correlation with MW (Figure 11a–f). Specifically, lignins with low to medium MW produced porous HCs, whereas high-MW lignin resulted in non-porous structures, reflected in its minimal SSA (0.71 m^2^ g^−1^, Table 3). The medium-MW HC carbonized at 700 °C displayed optimal features for Na storage, including balanced graphitic ordering, expanded interlayer spacing, and a largely amorphous texture. In line with these findings, Chen’s team [105] applied molecular sieving to fractionate pine lignin by MW and similarly observed that increasing MW led to diminished porosity and lower SSA values (Table 3, Figure 11g,h).

The role of oxygen in precursor feedstocks is widely recognized as pivotal in shaping the microstructure of HCs, primarily due to its significant influence on carbonization dynamics [31,55]. Chen and collaborators [105] systematically investigated the effects of polar O-containing functional groups on the structural and electrochemical performance of LHCs. They found that lignin dissolved in ethanol, rich in polar functionalities, undergoes vigorous pyrolysis, generating large open pores that fail to transition into closed pore structures. This high polarity also led to increased interlayer spacing and SSA. However, the study emphasized that a moderate degree of polarity and alcohol hydroxyl content yielded optimal microstructure and Na storage, with a closed P_v_, expanded interlayer spacing, and a capacity of 314 mAh g^−1^ (Table 3). The O-containing functional groups play a pivotal mechanistic role in determining both the structural evolution and Na-storage behavior of LHCs. During carbonization, hydroxyl, carbonyl, and carboxyl groups undergo progressive deoxygenation, releasing volatile species such as CO, CO_2_, and H_2_O that locally activate the C matrix and promote the development of micropores and enlarged interlayer spacing. These transient activation processes also facilitate partial graphitic rearrangement while introducing structural defects that serve as additional adsorption sites for Na^+^ ions. Residual O-atoms that remain bonded to the C framework (e.g., as C-O-C, C=O, or COOH) can modulate the electronic structure, enhance surface wettability, and contribute to pseudocapacitive charge storage at higher potentials. However, an excessive O-concentration tends to increase irreversible reactions with the electrolyte, leading to thick SEI formation and reduced ICE. Therefore, an optimal balance between O-content and C ordering is crucial: moderate O-functionalities are beneficial for achieving high reversible capacity and rate capability, whereas over-oxidized precursors often yield poorly conductive, unstable carbons. This mechanistic understanding underscores the importance of controlled feedstock pretreatment and fine-tuned carbonization protocols for tailoring O-chemistry in high-performance LHC anodes. Supporting this, Song et al. [119] reported that a reduction in overall O-content, while maintaining sufficient crosslinking, promoted the formation of pseudo-graphitic domains and increased closed P_v_ while lowering SSA [119]. In a subsequent study, Song’s group [120] showed that feedstocks rich in carbonyl groups led to enhanced interlayer spacing and reduced SSA, accompanied by smaller, thinner graphite-like crystals. Further insights from Tang and coworkers [118] revealed that specific oxygenated groups influence microstructure distinctly: ethers, carboxyls, and esters foster C-O-C bonding and disordered C with closed pores, whereas anhydrides, ketones, and exocyclic hydroxyls tend to support ordered structures with minimal nanoporosity (Figure 11i).

Inorganic impurities such as K, Ca, Na, and Si, which are naturally present in lignocellulosic feedstocks, exert significant influence on the structural evolution and Na storage behavior of HCs [25]. Ghimbeu’s group [89] reported that these inorganic species, when retained during pyrolysis, facilitate the development of LHCs with elevated SSA. Complementing this, Susanti and colleagues [106] demonstrated that pre-carbonization ash removal via washing enhances graphitization while concurrently reducing SSA. Their findings also revealed that while ashes contribute to the generation of additional porosity and structural defects, they exert limited impact on the formation of graphitic domains (Table 3). Notably, HCs produced via acid pretreatment exhibited excellent electrochemical properties, including a reversible capacity of 317 mAh g^−1^ and a plateau capacity of 244 mAh g^−1^. However, their study also emphasized that post-carbonization treatments are largely ineffective in altering HC structure, likely due to the persistent nature of embedded inorganic residues.

**Figure 12:**
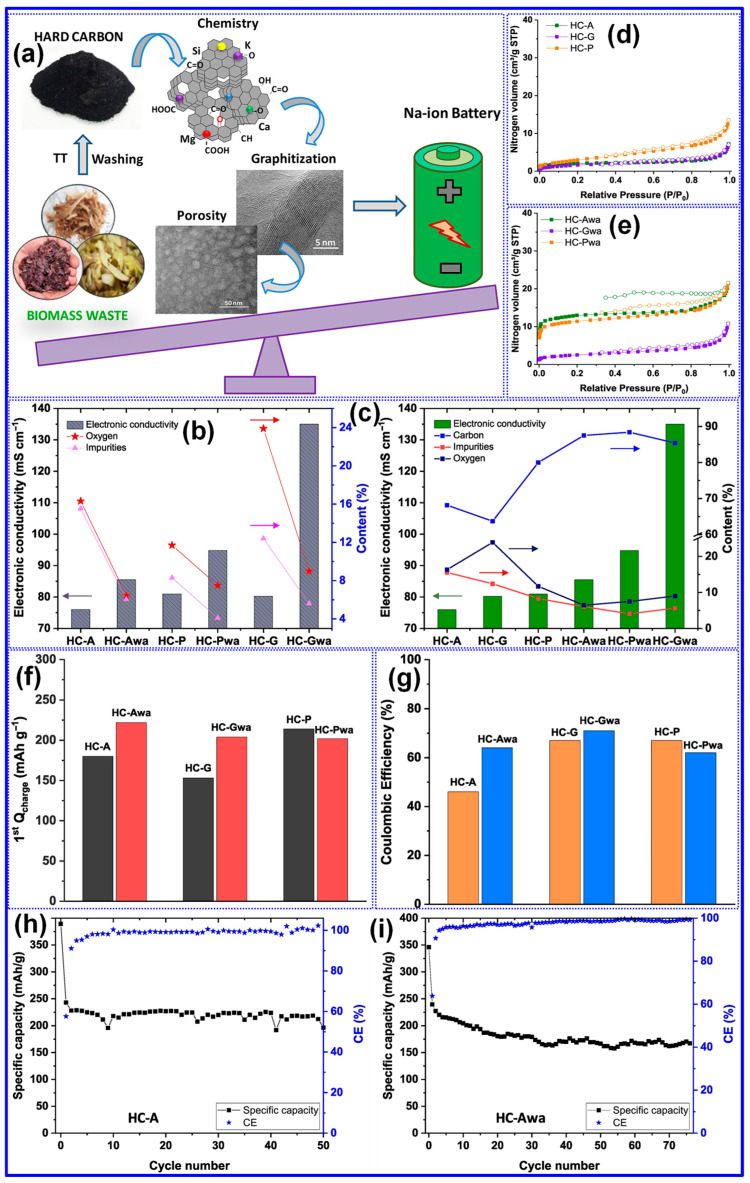
Impact of ash (inorganic elements) on the structure and Na storage performance of HCs. (**a**) Preparation process. Impact of ash on (**b**,**c**) electronic conductivity, (**d**,**e**) SSA, and (**f**–**i**) Na storage performances. Adapted from [121]. Copyright 2020, Elsevier B.V.

Contrary to previous reports, Beda’s group [121] reported divergent results regarding the effect of post-washing on the structure and electrochemical behavior of HCs. Their analysis revealed that most inorganic residues (ashes) are efficiently eliminated through washing, which in turn led to increased P_v_, SSA, and electronic conductivity. Concurrently, reductions were observed in interlayer spacing, O-content, and defect density (Figure 12a–e). These structural modifications correlated with enhancements in electrochemical properties, including both reversible capacity and ICE (Figure 12f–i). Nevertheless, the authors emphasized that such improvements were partially counteracted by adverse structural effects, most notably, the reduction in interlayer spacing and increase in SSA. Moreover, they reported that water washing prior to carbonization resulted in minimal structural or electrochemical change, which stands in contrast to the findings of Ghimbeu’s group [89]. A promising alternative, as reported in [122], is the two-step carbonization approach wherein ashes are removed following an initial pyrolysis step. Subsequent HCl washing and high-temperature carbonization not only ensure more complete ash elimination but also induce additional lattice defects, ultimately enhancing Na storage performance [122]. These inconsistent outcomes underscore the potential impact of ash composition and removal strategy, necessitating further systematic investigations to clarify the mechanisms by which inorganic residues influence the structure–property relationships of HCs.

The morphology of HC plays a pivotal role in determining its electrochemical performance [123,124]. Given that morphology is largely inherited from the precursor, it is classified as a feedstock-related parameter [7]. Morphological features influence key factors such as electron/ion transport kinetics and SSA, which collectively govern electrochemical behavior [15]. For instance, nanospheres contribute to shorter ion diffusion paths, higher packing density, and improved structural integrity, thereby enhancing ICE and rate capability. Nanowires improve electrolyte accessibility and ion transport, whereas nanosheets offer increased SSA and continuous electronic pathways. Additionally, 3D porous architectures facilitate effective ion diffusion [112,125]. The inherent polymeric structure and good solubility of lignin make it particularly amenable to morphology modulation. Wang’s team [107] fabricated LHCs with diverse morphologies and compared their structural and electrochemical characteristics (Table 3). Nonetheless, it should be noted that changes in morphology are often accompanied by variations in other lignin-derived properties, which may confound direct correlations.

**Figure 13:**
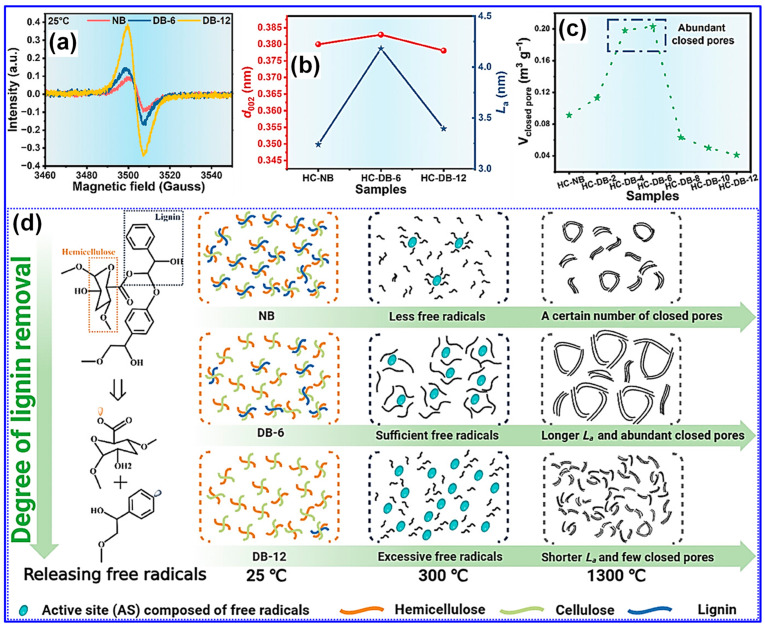
(**a**) Electron paramagnetic resonance (EPR) spectra of various precursors. (**b**) d_002_ and L_a_ values and (**c**) closed P_v_ of various HCs. (**d**) Mechanistic insights into free radical-driven microstructural evolution in HCs. Adapted from [126]. Copyright 2024, Wiley VCH.

Beyond morphology, the concentration of free radicals within lignin feedstocks plays a crucial role in determining the microstructural evolution of LHCs [126,127]. Wang and colleagues [126] introduced a method to control closed pore formation by tuning the free radical content through selective delignification using ClO_2_, which cleaves β-O-4 linkages. As the delignification duration increased, so did the concentration of free radicals (Figure 13a). Following carbonization at 1300 °C, the microcrystalline domain size (L_a_) and closed P_v_ first increased with free radical content and then declined, while the interlayer spacing (d_002_) remained relatively constant (Figure 13b,c). Moderate levels of free radicals were found to facilitate micropore formation and defect minimization [126]. Mechanistically, radicals enhance pyrolysis and act as intrinsic pore-forming agents, while also promoting microcrystal growth and aggregation, contributing to closed pore formation at elevated temperatures (Figure 13d). Conversely, excessive radical presence led to defect proliferation and hindered pore closure. A subsequent study by the same group employed an activation-based optimization of radical levels, further validating free radical tuning as an effective microstructure engineering strategy for LHCs [127].

This section has reviewed recent progress in elucidating how lignin feedstock characteristics influence the microstructure and electrochemical behavior of LHCs. Despite these advances, the intrinsic complexity of lignin continues to present challenges. Current understanding is largely based on comparative analyses of different lignin sources. However, these sources often differ concurrently in multiple attributes, such as MW, O-content, chemical structure, and inorganic (ash) content. For instance, lignins with varying MWs frequently also differ in O-content, while extraction techniques simultaneously alter ash levels and molecular profiles. As a result, decoupling the effects of individual variables on LHC structure and performance remains difficult.

#### 4.2.2. Operating Parameters

Carbonization temperature is a critical parameter governing the structural evolution and electrochemical performance of LHCs [58,108]. Optimizing this temperature is essential for achieving desirable material properties. As summarized in Table 3, increasing the carbonization temperature generally enhances the lateral crystallite size (L_a_) and stacking height (L_c_), while reducing interlayer spacing, structural defects (I*_D_*/I*_G_*), SSA, and P_v_. Correspondingly, the ICE typically increases with temperature. In contrast, specific capacity follows a non-linear trend, rising initially, peaking within 1200–1400 °C, and then declining. Similarly, cycling stability improves with elevated carbonization temperatures, but these trends hold only within a defined temperature window, beyond which deviations may occur.

Wu’s group [104] systematically studied the structural evolution of LHCs subjected to pyrolysis temperatures ranging from 500 to 1000 °C. Their results indicated that the interlayer spacing expands as temperature increases up to 700 °C, followed by a contraction at higher temperatures. Similarly, defect density rises and peaks near 800 °C before declining. These observations are explained by the dominance of two thermally activated processes: at 500–800 °C, depolymerization, fragmentation, and aromatization predominate, yielding highly disordered C structures with expanded spacing and increased defects; beyond 800 °C, graphitization takes over, reducing disorder and compressing interlayer distances.

Beyond the influence of carbonization temperature, the surrounding atmosphere plays a pivotal role in shaping the structural and electrochemical characteristics of LHCs. Marino’s group [110] synthesized three LHC samples via pyrolysis at 1000 °C for 6 h under different atmospheres: N_2_, Ar, and Ar containing 5% H_2_. Structural comparisons revealed negligible differences between the N_2_- and Ar-derived HCs, aside from a lower SSA observed in the Ar sample, likely attributable to the inertness of Ar relative to the slightly reactive N_2_ [17]. In contrast, the HC carbonized under an Ar/H_2_ reducing environment displayed similar crystallite dimensions, interlayer spacing, and defect levels, yet exhibited the largest SSA and closed P_v_. Electrochemical evaluations indicated comparable performance between N_2_ and Ar samples, while the Ar/H_2_-derived HC showed diminished capacity and ICE. At higher carbonization temperatures (1200–1400 °C), the divergence in structural features between HCs produced under N_2_ and Ar became more evident, likely due to the enhanced oxidizing potential of N_2_ at elevated temperatures [17].

Besides, Garcia-Negron’s team [109] systematically explored the influence of water vapor concentration during carbonization on the structural evolution of LHCs. Kraft softwood lignin was pyrolyzed at 1000 °C under a N_2_ atmosphere with controlled water vapor levels ranging from 0 to 105 g cm^−3^. While the presence of water vapor exerted negligible influence on graphitic attributes, such as crystallite size and interlayer spacing, it had a pronounced effect on pore development. Specifically, increased moisture content resulted in larger pore sizes, greater P_v_, and elevated SSA. These effects are attributed to the occurrence of the water–gas shift reaction at temperatures exceeding 800 °C.

**Figure 14:**
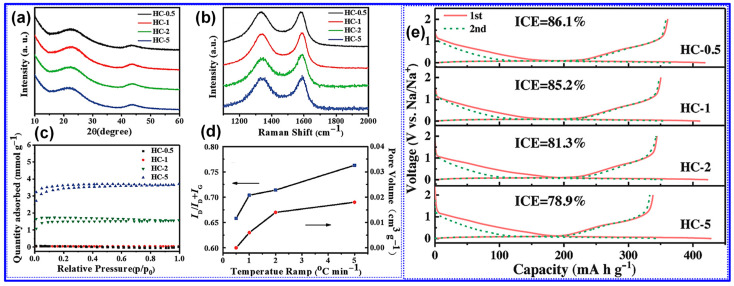
Evaluation of heating rate-dependent properties in HCs. (**a**) XRD, (**b**) Raman, (**c**) N_2_ adsorption–desorption isotherms, (**d**) I*_D_*/I*_G_* and P_v_, and (**e**) the discharge–charge profiles in Na half-cell. Adapted from [128]. Copyright 2018, Wiley VCH.

Xiao’s team [128] systematically examined the influence of heating rate on the structural evolution and electrochemical performance of HCs synthesized at 1300 °C. As illustrated in Figure 14a–e and detailed in Table 3, a reduced heating rate resulted in decreased interlayer spacing, P_v_, SSA, and defect density, alongside increased crystallite size and thickness of graphite-like domains. These microstructural enhancements corresponded to improvements in both ICE and specific capacity. The authors attributed these findings to the more efficient release of gaseous byproducts at lower heating rates, which inhibits excessive pore formation and facilitates graphitic ordering.

Similarly, Guo and colleagues [111] also examined the influence of heating rates on camphor-derived HCs, revealing both consistencies and divergences compared to the findings of Xiao et al. [128] Notably, Guo’s group observed that lower heating rates led to increased interlayer spacing and decreased crystallite size and thickness, opposite to the trends reported by Xiao. Furthermore, they highlighted the role of shielding gas flow rates, noting that higher flow rates reduce the residence time of volatile gases, limiting their interaction with the C matrix and thereby reducing both P_v_ and SSA [17]. Additional variables, including carbonization duration, initial pyrolysis temperature in two-step syntheses, and the nature and concentration of activating agents, also affect the microstructure and performance of LHCs. Nonetheless, these factors remain underexplored, and further investigation is required to draw firm conclusions.

## 5. Optimization Techniques of LHCs

**Figure 15:**
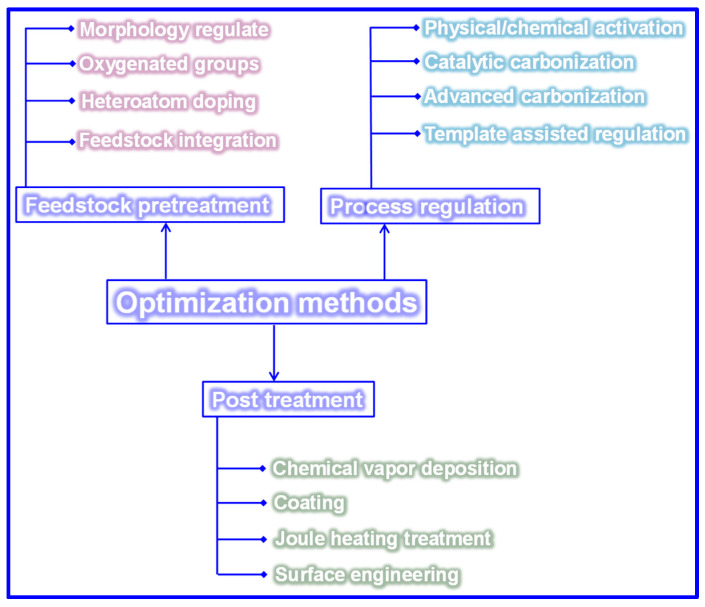
Optimization techniques of LHCs.

Carbonization remains a widely adopted technique for fabricating LHCs due to its simplicity. However, the direct carbonization approach often falls short in achieving the desired microstructure and Na storage performance, as shown in Table 3. Since the structural and electrochemical characteristics of LHCs are largely governed by the nature of the feedstock and processing parameters, strategies such as feedstock pretreatment, controlled processing, and post-synthesis modification are frequently employed to enhance these characteristics (Figure 15).

### 5.1. Feedstock Pretreatment Techniques

A strong correlation between the structural and compositional characteristics of feedstocks and the resulting microstructure of HCs has been extensively reported in the literature [129]. Concurrently, numerous studies have validated the effectiveness of feedstock pretreatment in enhancing the Na storage performance of HCs. Common pretreatment strategies include morphology control, regulation of O-containing functional groups, heteroatom doping, and feedstock integration.

#### 5.1.1. Morphology Tuning

Lignin exhibits excellent morphological adaptability, attributed to its polymeric structure and solubility. A wide range of studies have confirmed that C materials derived from lignin can be fabricated into various morphologies such as spheres, fibers, sheets, and 3D porous foams [130,131]. Notably, C spheres stand out as ideal anode candidates for SIBs, offering advantages such as high packing density, low surface area-to-volume ratio, reduced Na^+^ diffusion distance, and limited defect density. These features collectively suppress SEI formation and promote improved structural integrity and Na storage capacity [131,132].

**Figure 16:**
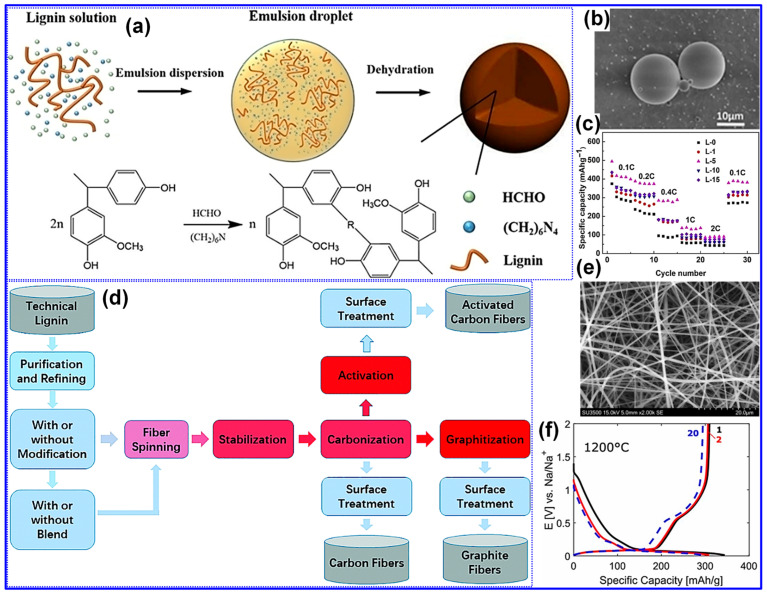
(**a**) Schematic of lignin-derived C sphere synthesis, (**b**) SEM micrograph of L-5 C sphere, and (**c**) rate performance of prepared C spheres from 0.1 to 2 C. Adapted from [133]. Copyright 2018, Wiley VCH. (**d**) Typical manufacturing and (**e**) SEM micrograph of lignin-derived C fibers (LCFs) prepared by electrospinning followed by carbonization at 1000 °C. (**f**) Charge/discharge curves of LCF carbonized at 1200 °C under 30 mA g^−1^. Adapted from [134]. Copyright 2017, Royal Society of Chemistry.

Typically, lignin-derived spheres are synthesized through techniques such as reversed-phase polymerization, solvent exchange, spray drying, or hydrothermal processing, succeeded by high-temperature carbonization to produce HC spheres (HCSs). As a representative example, Yu and colleagues [133] demonstrated the preparation of lignin-based HCS via reversed-phase polymerization, as depicted in Figure 16a. In this method, lignin was combined with HMTA and formaldehyde in an aqueous medium to form an emulsion, which was subsequently dispersed within olive oil to establish a reversed-phase suspension polymerization environment. Upon water evaporation, lignin-derived microspheres were obtained and subjected to carbonization at 1300 °C to yield HCS. SEM analysis (Figure 16b) verified the formation of uniform spherical morphologies, while electrochemical characterization (Figure 16c) revealed outstanding Na storage capabilities, including a specific capacity of 393 mAh g^−1^ and an ICE of 79.6%. Moreover, the same research group successfully fabricated lignin-based HCS utilizing a double-solvent evaporation coupled with resinification [125].

Attributable to its nanospherical configuration and distinctive structural attributes, the optimized HCS exhibits a specific capacity of 347 mAh g^−1^ alongside an ICE of 74%. Fan’s group [113] synthesized N-doped LHC microspheres via a hydrothermal method, yielding structures with accelerated Na-ion adsorption and intercalation kinetics and demonstrating remarkable electrochemical properties with a capacity of 374 mAh g^−1^ and an ICE of 85%. In a separate investigation, Li and co-workers [112] employed spray drying using sodium lignin sulfonate as the sole C precursor to fabricate HC microspheres characterized by expanded interlayer spacing, low defect concentration, and a reduced SSA (11.89 m^2^ g^−1^). These features collectively contributed to a high reversible capacity of 339 mAh g^−1^ and an outstanding ICE of 88.3%.

A variety of spinning methodologies, including melt-spinning, wet-spinning, dry-spinning, and electrospinning, are routinely utilized for the production of lignin-derived fibers [134]. To transform these precursor fibers into CFs, stabilization and carbonization are indispensable post-processing steps, as illustrated in Figure 16d. Furthermore, additional treatments such as activation and graphitization may be employed to refine the microstructure and enhance the functional properties of the resulting CFs.

**Figure 17:**
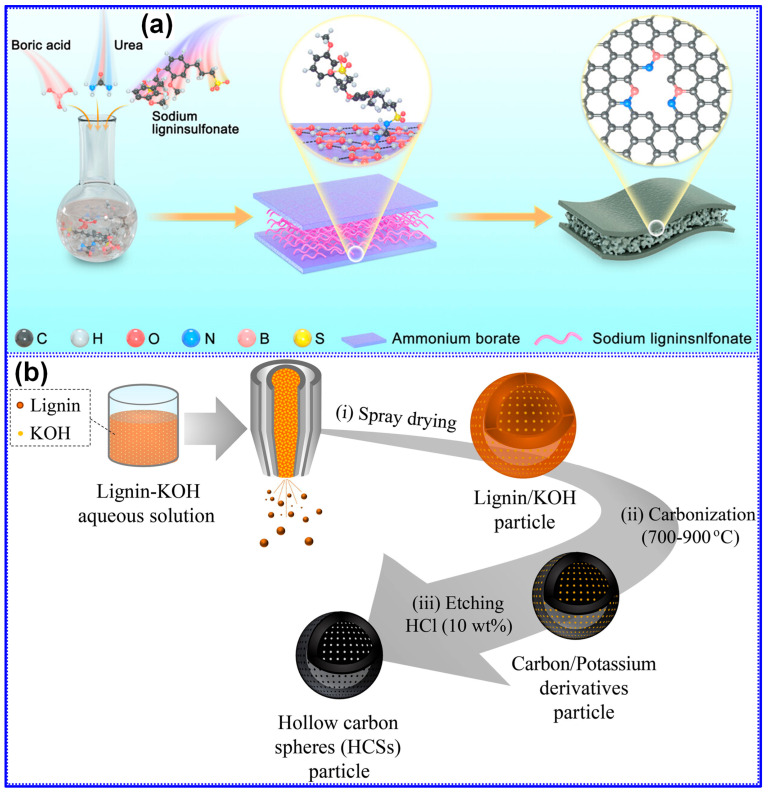
Schematic diagram of the fabrication of lignin-based (**a**) C NSs. Adapted from [130]. Copyright 2024, Wiley VCH. (**b**) Hollow C spheres. Adapted from [136]. Copyright 2021, Elsevier B.V.

Peuvot’s team [108] synthesized LCFs via electrospinning, followed by high-temperature carbonization in the range of 800–1700 °C, as illustrated in Figure 16e. Their investigation demonstrated that carbonization at 1200 °C produced HC fibers exhibiting promising performance as SIB anodes, with a specific capacity of 310 mAh g^−1^ and an ICE of 89% (Figure 16f). In a related study, Zhao and co-workers [135] fabricated flexible lignin/PAN CFs through electrospinning, pre-oxidation, and subsequent carbonization. By modulating the cooling rate during the pre-oxidation stage, they effectively tailored the nanofiber’s chemical structure, achieving a reversible capacity of 207 mAh g^−1^ with excellent cycling durability. Beyond fibrous morphologies, lignin-derived C materials have also been engineered into alternative architectures, such as nanosheets and porous C. For instance, Jiang’s group [130] reported a nano-sandwich structured porous C enriched with pyridinic N-B species, fabricated via a self-assembly template approach (Figure 17a). Similarly, Cao’s team [136] prepared porous hollow C from lignin through a spray-assisted method combined with KOH activation during carbonization (Figure 17b). Despite the marked improvements in electrochemical performance achieved through morphology engineering, the inherent complexity and associated costs of these synthesis routes pose significant challenges to large-scale commercial deployment. Furthermore, although the morphology of HC materials profoundly affects electrode fabrication, rate performance, and cycling stability, comprehensive systematic investigations into these correlations remain scarce.

#### 5.1.2. Oxygenated Group Tuning

Oxygenated functional groups exert a profound influence on lignin’s crosslinking behavior, pyrolysis kinetics, and overall reactivity, thereby determining both the resultant yield and microstructural characteristics of LHCs [137,138]. Accordingly, the deliberate regulation of these oxygenated moieties has emerged as a pivotal pretreatment strategy to refine the structural features and electrochemical properties of LHCs.

**Figure 18:**
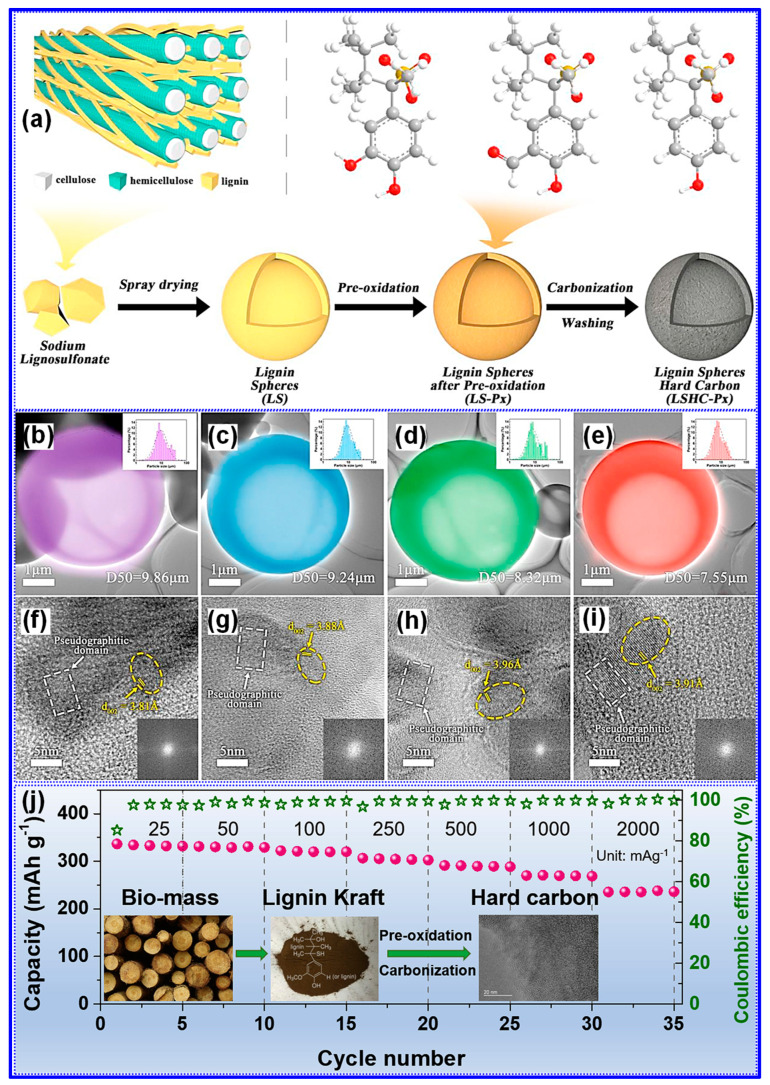
(**a**) Schematic of LHC synthesis via pre-oxidation. TEM micrographs of LHCs pre-oxidized at various temperatures: (**b**,**f**) room temperature, (**c**,**g**) 150, (**d**,**h**) 200, and (**e**,**i**) 250 °C. Adapted from [120]. Copyright 2021, Elsevier B.V. (**j**) Schematic of LHC fabrication via pre-oxidation at 200 °C and subsequent carbonization at 1350 °C, along with its electrochemical characteristics. Adapted from [138]. Copyright 2020, Elsevier B.V.

Among the available methods, pre-oxidation remains the most prevalent approach for modulating lignin’s O-functionalities. Du’s team [120] employed this strategy to synthesize LHCs (Figure 18a) and conducted a comprehensive investigation of the underlying mechanisms and structural implications. Their results revealed that three principal oxygenated species, hydroxyl, carbonyl, and carboxyl groups, are formed during pre-oxidation, with their relative abundance governed by temperature. Specifically, at 150 °C, demethylation predominates, resulting in hydroxyl formation; at 200 °C, carbonyl groups become most abundant; and at 250 °C, carboxyl groups dominate. The introduction of these O-functionalities enhances lignin crosslinking, yielding increased interlayer spacing and a more disordered C nanotexture (Figure 18b–i). Notably, their findings concluded that carbonyl groups exert the most favorable influence, with 200 °C identified as the optimal pre-oxidation temperature.

Similarly, Lin and colleagues [138] employed low-temperature pre-oxidation to selectively enrich carbonyl functionalities within lignin. This treatment enhanced crosslinking and effectively inhibited the alignment of graphitic layers during subsequent carbonization. The resultant LHC, prepared by pre-oxidation at 200 °C and carbonization at 1350 °C, demonstrated increased interlayer spacing, a high specific capacity of 307 mAh g^−1^ at a current density of 25 mA g^−1^, and superior cycling stability and rate capability (Figure 18j). In a related study, Zheng and co-workers [139] concentrated on maximizing C yield through pre-oxidation of lignin. Their findings revealed a non-linear dependence of yield on pre-oxidation temperature. By incorporating a gas-phase removal-assisted aqueous washing step followed by sequential carbonizations at 1000 and 1400 °C, they fabricated a cross-linked oxidized LHCs exhibiting an exceptional Na storage capacity of 359 mAh g^−1^.

In summary, modulating the oxygenated functional groups within lignin can markedly improve its crosslinking capacity, enhance carbonization efficiency, expand the interlayer distance in HC, and inhibit graphitic ordering, thereby fostering a more disordered microstructure. These structural transformations are positively correlated with enhanced capacity and rate capability in LHCs. Nevertheless, it is crucial to acknowledge that increased O-content may also lead to higher SSA and a greater abundance of surface O-containing groups, both of which can negatively impact ICE and cycling stability [140]. Additionally, due to the heterogeneous nature of lignin across various biomass sources, the effectiveness of pre-oxidation treatments may not be universally transferable.

#### 5.1.3. Heteroatom Doping

Heteroatom doping is a well-established method for tailoring the microstructure of HCs and improving their electrochemical performance [18,23]. A substantial body of research has shown that the introduction of heteroatoms into the C matrix induces favorable modifications such as expanded interlayer spacing, increased defect density, improved electronic conductivity, enhanced surface wettability, accelerated ion transport, and lower reaction energy barriers. Collectively, these changes enhance Na storage performance [141,142]. Nitrogen (N), sulfur (S), phosphorus (P), and boron (B) are the most commonly used dopants, with N being particularly prevalent due to its advantageous chemical and electronic characteristics [80,143]. While oxygen (O) is frequently present in HCs, it is generally intrinsic rather than introduced through deliberate doping and is thus excluded from this discussion. Heteroatom incorporation typically occurs via one of three routes [142,144]: (1) carbonization of inherently heteroatom-rich feedstocks, (2) co-carbonization with heteroatom-containing additives, or (3) post-carbonization treatment. For LHCs, the first two strategies are especially pertinent, aligning with the feedstock pretreatment stage of synthesis.

**Figure 19:**
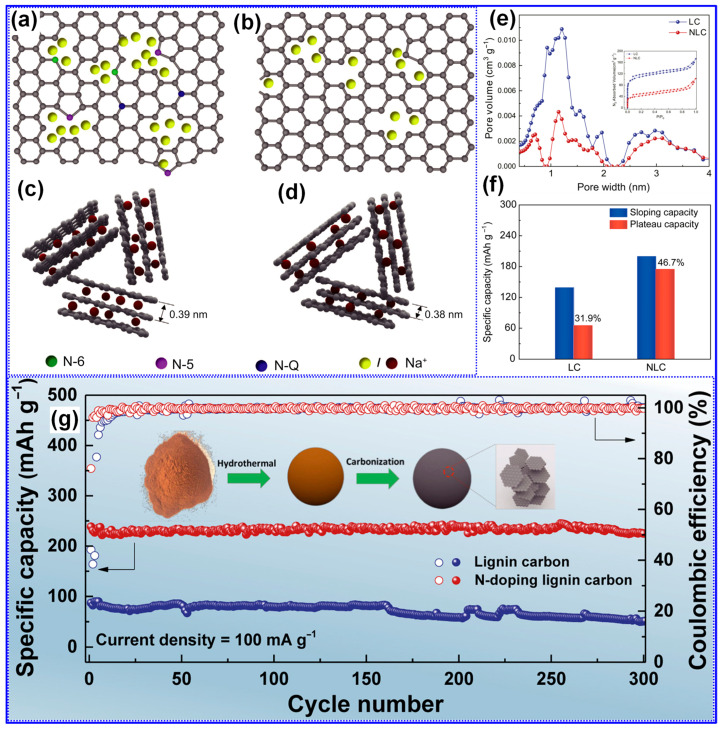
(**a**–**d**) Schematic representation of N-doped LHC and a comparative analysis of Na^+^ storage behavior between N-doped and undoped LHC. (**e**) Pore size distribution, (**f**) capacity contributed by the plateau and sloping regions, and (**g**) cycling performance of N-doped and undoped LHC. Adapted from [113]. Copyright 2021, Elsevier B.V.

Nitrogen is the most extensively utilized heteroatom for doping HCs [2,145]. Incorporation of N into the C matrix has been shown to enhance electrical conductivity, increase surface wettability, expand interlayer spacing, and introduce additional electrochemically active sites [18]. In a representative study, Fan and colleagues [113] synthesized N-doped LHC employing 3-aminophenol as the N source and lignin as the C precursor. The synthesis protocol included hydrothermal treatment at 250 °C for 12 h, followed by carbonization at 1100 °C for 2 h, resulting in an LHC material containing 2.76% N. N atoms were incorporated into the C lattice in the forms of pyridinic N (N-6), pyrrolic N (N-5), and quaternary N (N-Q), as depicted in Figure 19a–d. Compared to its undoped counterpart, the N-doped LHC demonstrated larger interlayer spacing and a slightly reduced SSA, which together contributed to improved sloping and plateau-type Na storage capacities (Figure 19e–g). Consequently, the material exhibited a high reversible capacity of 374 mAh g^−1^ and an ICE of 85% at a current density of 25 mA g^−1^.

**Figure 20:**
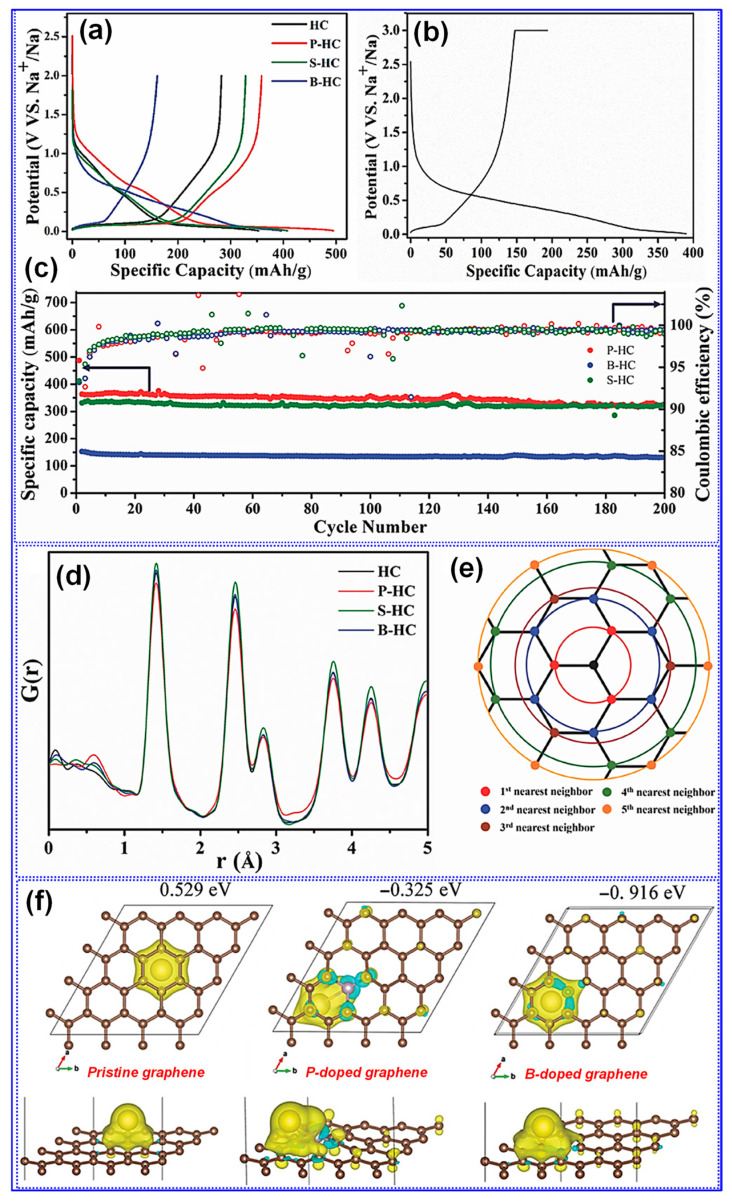
(**a**–**c**) The Na storage performances, (**d**,**e**) neutron total scattering and pair distribution function analysis, and (**f**) charge distribution curves of P-, S-, B- and un-doped HCs. Adapted from [148]. Copyright 2017, Wiley VCH.

Chen and colleagues [146] successfully synthesized N-doped LHC with superior rate capability by carbonizing alkaline lignin in the presence of melamine and urea. The resulting material delivered a reversible capacity of 320.5 mAh g^−1^ at 0.1 C and retained 138.7 mAh g^−1^ at 5 C. This rate performance enhancement was primarily attributed to the increased porosity and defect density introduced through N doping. Beyond N, heteroatom doping with B, P, and S has also been investigated [2,147]. For example, Li and co-workers [148] synthesized B-, P-, and S-doped HCs via co-carbonization of a C precursor with H_3_PO_4_, H_2_SO_4_, and H_3_BO_3_ at 1100 °C for 5 h. Their results indicated that P and S dopants expanded interlayer spacing via steric effects, which enhanced plateau capacity. Conversely, P and B doping increased the defect density, thereby improving the sloping capacity (Figure 20a–c). DFT calculations confirmed that P and B doping within the graphene lattice yielded favorable Na^+^ binding energies (BEs); however, the B-doped material exhibited stronger binding than the P-doped counterpart, resulting in higher irreversible capacity (Figure 20d–f). Despite these promising outcomes, reports of LHCs singly doped with heteroatoms other than N remain scarce [142], mainly due to the increased complexity associated with their synthesis.

**Figure 21:**
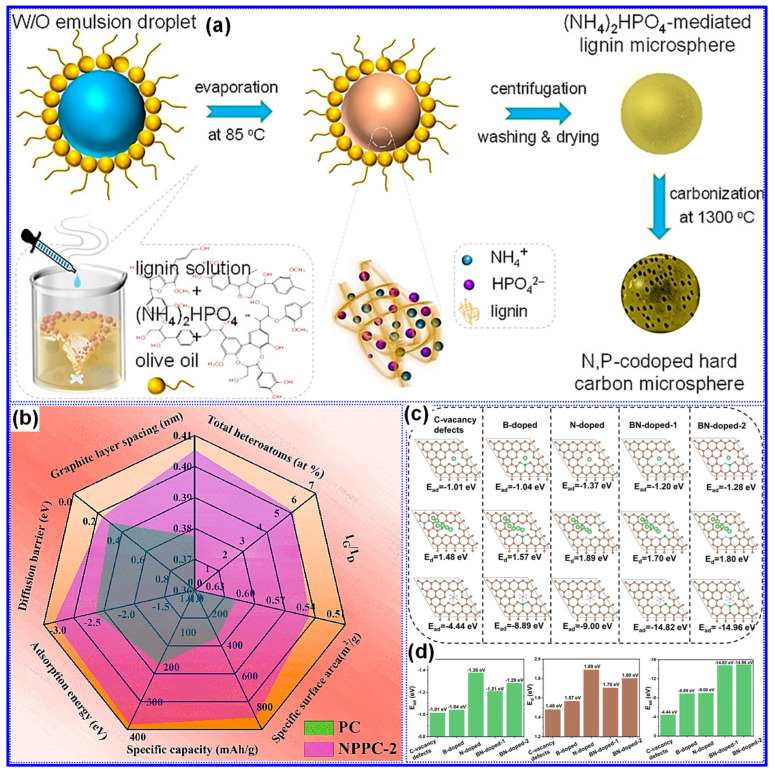
(**a**) Schematic diagram depicting the synthesis process of N,P co-doped LHCs. Adapted from [150]. Copyright 2021, Wiley VCH. (**b**) Comparative analysis of structural characteristics and Na storage behavior between un-doped and N,P co-doped HCs. Adapted from [151]. Copyright 2021, Elsevier B.V. (**c**,**d**) Comparative analysis of adsorption and diffusion energies of doped and un-doped LHCs. Adapted from [130]. Copyright 2024, Wiley VCH.

Compared to single-heteroatom doping, multi-heteroatom co-doping has attracted increasing attention due to its potential to leverage the synergistic effects of individual heteroatoms [147,149]. For example, Zhang and co-workers [150] prepared N,P co-doped LHCs using ((NH_4_)_2_HPO_4_ as a dual source of N and P via an emulsion–solvent evaporation technique (Figure 21a). Carbonization at 1300 °C for 2 h enabled effective incorporation of both elements into the C matrix, resulting in LHCs with significantly improved reversible capacity and cycling performance compared to their undoped analogues. In a comparative study, Chen and co-workers [151] found that N,P co-doping not only increased the SSA and defect density but also expanded the interlayer spacing, reduced Na^+^ diffusion barriers, and enhanced adsorption energies, collectively contributing to superior Na storage performance (Figure 21b). More recently, we synthesized N,B co-doped LHCs enriched in pyridinic N-B structures using urea and boric acid through a self-assembly template strategy (Figure 17a) [130]. Beyond conventional co-doping effects, our investigation focused on the role of pyridinic N-B configurations (Figure 21c,d), which were found to further raise the BE and diffusion barrier for Na^+^ ions, exceeding the impact of general N,B co-doping.

Heteroatom doping is widely recognized as a viable approach for engineering the microstructure of LHCs and improving their Na storage performance. Nonetheless, several critical challenges persist. Firstly, the associated increase in SSA and defect density can detrimentally affect the ICE and long-term cycling stability. Secondly, the high synthesis temperatures typically required (>1000 °C) may limit the retention or effectiveness of dopants. Thirdly, the doping process introduces additional complexity and cost and may raise environmental concerns depending on the dopant precursors used. Finally, heteroatom doping can obscure the fundamental Na storage mechanisms, complicating the rational design of high-performance LHCs.

#### 5.1.4. Feedstock Integration

**Figure 22:**
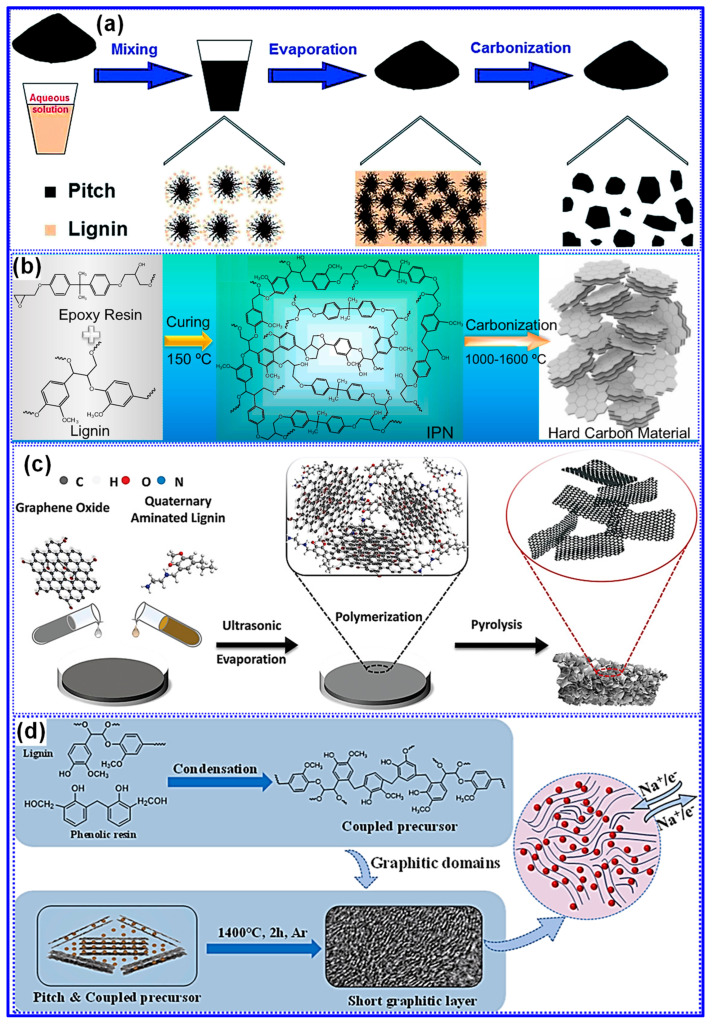
Illustration of the integration strategy between lignin and complementary precursors: (**a**) Pitch. Adapted from [158]. Copyright 2016, Royal Society of Chemistry. (**b**) Epoxy resin. Adapted from [159]. Copyright 2018, Elsevier B.V. (**c**) GO. Adapted from [156]. Copyright 2023, Wiley VCH. (**d**) Phenolic resin and pitch. Adapted from [155]. Copyright 2024, Elsevier B.V.

Each C precursor exhibits distinct structural and chemical traits, making it advantageous to integrate lignin with complementary materials to engineer LHCs with heterogeneous architectures and enhanced Na storage capabilities [152,153]. Studies have demonstrated that co-carbonization of lignin with other precursors can produce synergistic effects, significantly improving electrochemical performance [114,154]. Typically, this approach results in one of three heterostructure types: hard-hard, hard-soft, or hard-nano [155,156,157]. For example, Li and collaborators [158] developed a series of hard–soft C heterostructures by co-carbonizing lignin and pitch at various mass ratios and temperatures (Figure 22a). Their results indicated that lignin suppresses pitch graphitization, thereby increasing interlayer spacing, while pitch reduces the SSA of the LHCs. Under optimal conditions, the composite delivered a high reversible capacity of 254 mAh g^−1^ and an ICE of 82%.

In contrast, Zhang’s group [159] developed “hard–hard” heterostructured LHCs by co-pyrolyzing lignin with epoxy resin (Figure 22b). Through careful adjustment of the precursor mass ratio and the carbonization temperature, the resulting materials exhibited tunable interlayer spacing and defect densities. Remarkably, these heterostructures presented larger interlayer spacings than either of the single-component carbons derived solely from lignin or epoxy resin, implying that chemical interactions occurred during pyrolysis, as opposed to simple physical mixing. Under the optimized condition of a 1:1 lignin-to-epoxy ratio and carbonization at 1400 °C, the material delivered an impressive reversible capacity of 316 mAh g^−1^ and an ICE of 82%.

Furthermore, the integration of nanocarbons into LHCs has proven effective in enhancing electronic conductivity and mitigating defect formation, thereby boosting Na storage performance [160]. For instance, Zhong’s group [156] introduced graphene oxide (GO) into lignin precursors, which led to a “hard-nano” heterostructure featuring localized graphitic nanodomains embedded within the C matrix, an interlayer spacing of 0.42 nm in amorphous regions, and reduced defect density (Figure 22c). Similarly, a unique “hard–soft” heterostructure was developed by incorporating pitch-derived graphitic domains into a C matrix formed via reaction between lignin and phenolic resin [155]. Characterized by low SSA, efficient electron/Na^+^ transport, and enlarged interlayer spacing, this composite achieved a high ICE of 89% and delivered a notable capacity of 192.5 mAh g^−1^ at a high current density of 6 C (Figure 22d).

In addition to the studies discussed above, a variety of other feedstocks, including hemicellulose [161], cellulose [162], 3-Aminophenol [163], and PAN [164], have been successfully integrated with lignin to synthesize heterogeneous C. Owing to their straightforward processing and beneficial synergistic effects, such feedstock integration approaches are considered highly promising for enhancing the electrochemical performance of LHCs. Nonetheless, a deeper understanding of the underlying synergistic interactions and Na storage mechanisms in these heterogeneous C materials remains a significant challenge.

Overall, feedstock pretreatment has proven to be a highly effective and increasingly employed approach for engineering the microstructure of LHCs and enhancing their Na storage capabilities. While significant progress has been made in recent years, several fundamental challenges remain. Chief among these is the need to elucidate the correlations between the intrinsic physical and chemical characteristics of lignin and the resulting C microstructure. Such insights are essential for establishing a theoretical framework to guide pretreatment objectives and methodological selection. Furthermore, it is imperative that pretreatment strategies strike a balance between performance optimization, cost efficiency, and compatibility with large-scale LHC production processes.

### 5.2. Preparation Approach Tuning

As previously outlined, the preparation process is pivotal in dictating the microstructure and, consequently, the Na storage performance of LHCs. Traditional process optimization approaches have largely focused on adjusting parameters such as heating rate, carbonization temperature, and dwell time [165,166]. While refining these parameters remains fundamental, such measures alone are often inadequate to yield LHCs with superior electrochemical performance. Accordingly, recent advancements have introduced a suite of innovative techniques, encompassing physical and chemical activation, catalytic carbonization, advanced thermal processing, and template-assisted structural engineering, to more precisely modulate C structure and enhance Na storage efficiency.

#### 5.2.1. Chemical/Physical Activation

Chemical and physical activation techniques have long been utilized to develop porous architectures within C materials [167,168]. Although open porosity is generally regarded as detrimental for HCs in SIBs, activation was traditionally deemed non-essential for HC fabrication. Nonetheless, Wang and colleagues [169] revealed that nanopores formed during the activation stage can be effectively converted into closed pores through subsequent high-temperature carbonization. This process also enhanced the degree of microcrystalline order in the HCs. Building upon this concept, Zheng and co-workers [170] recently employed a CO_2_-etching method to introduce open porosity, which was then transformed into closed pores via elevated-temperature treatment. This approach yielded a HC material with an exceptional Na storage capacity of 487.6 mAh g^−1^ and an ICE of 90.56%.

**Figure 23:**
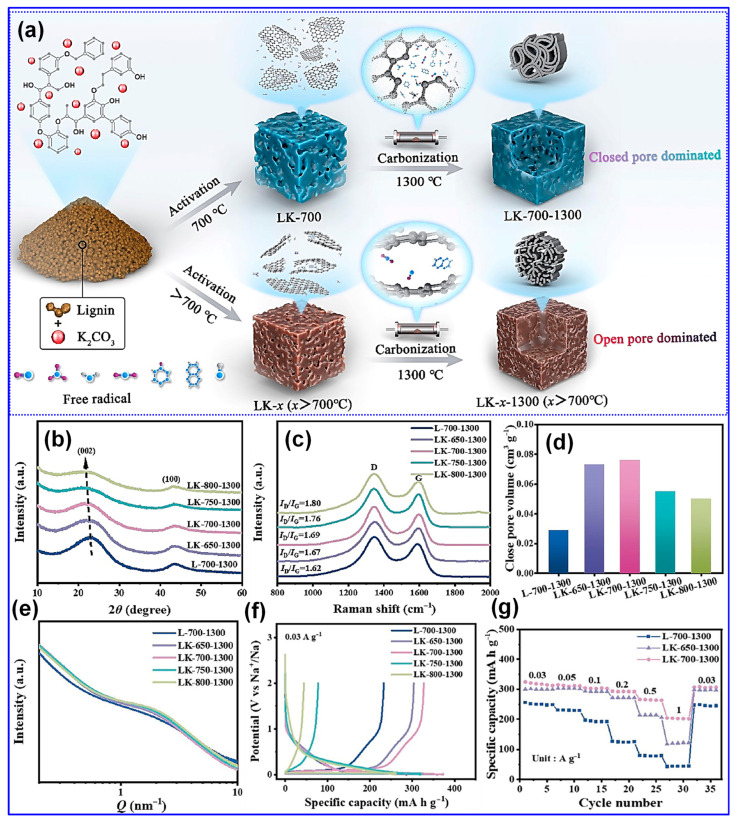
(**a**) Illustration of the activation-induced structural transformation and self-healing mechanism. (**b**) XRD, (**c**) Raman, (**d**) closed P_v_, (**e**) SAXS, (**f**) charge/discharge plots, and (**g**) rate performance of LHCs prepared with or without K_2_CO_3_ activation. Adapted from [127]. Copyright 2024, Elsevier B.V.

Although significant progress has been made, the underlying mechanism governing the transformation of open pores into closed ones remains inadequately understood. To address this knowledge gap, Ji and colleagues [127] conducted a detailed investigation into pore evolution in LHCs, employing K_2_CO_3_ as the activating agent (Figure 23a). By systematically varying the activation temperature and analyzing the resulting microstructures (Figure 23b–g), they discovered that the concentration of free radicals within the C matrix is a determining factor in the successful closure of open pores during high-temperature processing. Notably, activation temperatures exceeding 700 °C led to diminished radical concentrations, thereby inhibiting pore closure. Conversely, activation at the optimal temperature of 700 °C yielded the highest volume of closed pores and achieved a reversible capacity of 330.8 mAh g^−1^ at 0.03 A g^−1^.

**Figure 24:**
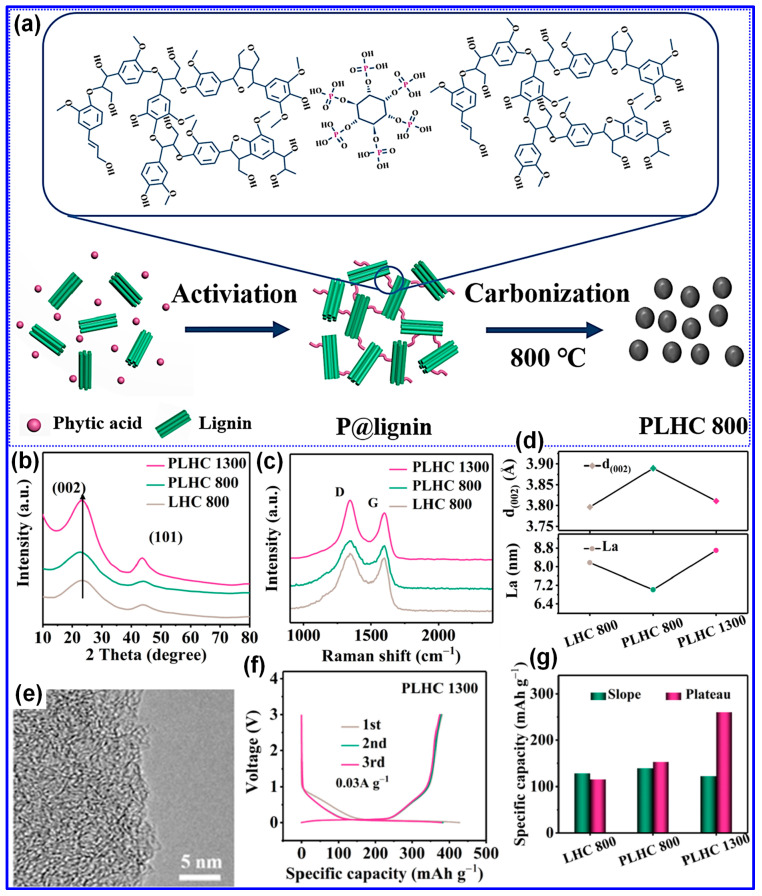
(**a**) Illustration of the synthesis pathway for LHCs using phytic acid as an activating agent. (**b**) XRD, (**c**) Raman, and (**d**) interlayer spacing and L_a_ values. (**e**) HR-TEM micrographs, (**f**) charge/discharge profiles of PLHC-1300, and (**g**) specific capacities of LHCs. Adapted from [171]. Copyright 2024, Wiley VCH.

In a recent investigation, He and colleagues [171] developed a phytic acid-assisted activation method combined with high-temperature annealing to synthesize LHC for SIBs (Figure 24a). The study demonstrated that phytic acid not only promotes the generation of numerous closed pores but also reduces the degree of microcrystalline ordering. As a result, the LHC exhibited a high reversible capacity of 380.3 mAh g^−1^ at 0.03 A g^−1^ (Figure 24b–g). Nevertheless, converting open pores into closed ones during subsequent high-temperature treatment remains challenging, often leading to elevated SSA and diminished ICE [172]. Additionally, the mechanisms underlying closed pore formation, especially their dependence on C precursors and pore-forming agents, are still not fully understood and require further in-depth research.

#### 5.2.2. Catalytic Carbonization

The intrinsic disorder and low graphitization of HC hinder its electrical conductivity, rate capability, and long-term cycling stability due to a high density of structural defects [173]. To overcome these challenges, metal-ion-catalyzed carbonization has been employed to incorporate graphitic nanoribbons into the amorphous C matrix. This hybrid architecture combines the benefits of both ordered and disordered C domains [174,175]. For instance, Yu’s team [176] successfully fabricated a heterogeneous HC featuring long-range ordered nano C networks via Fe-catalyzed carbonization. The Fe catalyst promoted the cleavage of sp^3^ C linkages, enabling graphene sheet reorganization and interlayer expansion at high temperatures. This architecture enhanced ion transport and electronic conductivity, delivering improved Na storage performance.

**Figure 25:**
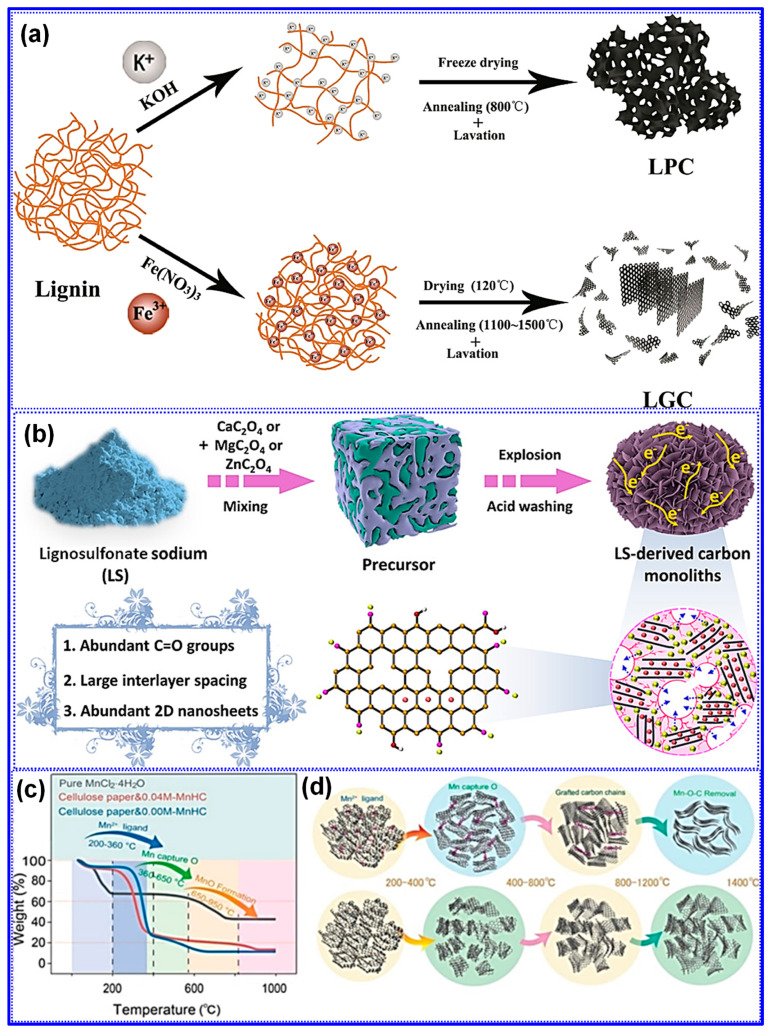
Pictorial representation of LHC fabrication by (**a**) Fe-ion-catalyzed carbonization. Adapted from [177]. Copyright 2020, American Chemical Society. (**b**) Metal oxalate-catalyzed explosion approach. Adapted from [178]. Copyright 2023, American Chemical Society. (**c**) TG curves of three samples with different Mn ion contents and (**d**) the schematic diagram of preparation of HC by Mn-ion-catalyzed carbonization. Adapted from [179]. Copyright 2023, Wiley VCH.

Likewise, Yang and colleagues [177] employed Fe-catalyzed carbonization to construct LHCs with extended graphitic domains embedded within an amorphous C framework (Figure 25a). Beyond Fe, other metals such as K and Ni have also demonstrated catalytic graphitization effects during carbonization and have been successfully utilized to form nanographitic structures in HC [173].

In addition to facilitating the formation of graphitic nanoribbons, catalytic carbonization has been shown to influence the distribution of O-containing functional groups, interlayer spacing, and defect density within HCs [178,179]. Wang’s team [178] developed an innovative metal oxalate-catalyzed “explosion” method to synthesize LHC materials enriched in carbonyl functionalities and exhibiting expanded interlayer spacing (Figure 25b). The underlying mechanism is attributed to the rapid decomposition of metal oxalates, which initiates a catalytic carbonization process that promotes demethylation of methoxyl groups and cleavage of carboxyl groups in lignin, leading to the formation of carbonyl-rich structures. Concurrently, the evolution of CO_2_ during pyrolysis etches the C matrix, increasing heteroatom incorporation and widening interlayer gaps. A comparative analysis of various metal oxalates (Ca, Mg, and Zn) revealed that their catalytic effects differ according to decomposition temperatures.

Zhao and collaborators [179] proposed a metal ion-assisted catalytic carbonization strategy that effectively modulates the graphitization degree while reducing defect concentration in HCs. Advanced characterization techniques revealed that Mn ions initially coordinate with oxygenated functional groups at low temperatures and subsequently catalyze the cleavage of sp^3^ C bridges at higher temperatures. This dual-stage process promotes the realignment of graphene layers, facilitating nanographitic domain growth and defect healing (Figure 25c,d). By adjusting the type and concentration of metal ions, the study demonstrated precise control over interlayer spacing, d_002_, L_a_, pore structure, graphitization degree, and electrochemical properties. For example, a concentration of 0.04 M Mn yielded HCs with extended graphitic domains, minimal defects, a reversible capacity of 336.8 mAh g^−1^, and an ICE of 92.05%. Building upon this concept, Zhou et al. [79] recently introduced a tandem catalytic carbonization technique that further enables fine-tuning of graphite domain size, interlayer distance, and closed pore formation. This method yielded HCs with long-range ordered graphitic structures (L_a_ = 5.31 nm), expanded d_002_ (0.389 nm), and rich closed porosity, driven by the temperature-responsive catalytic activity of Zn-based precursors.

In general, metal salts are commonly employed as catalysts in carbonization processes due to their ability to promote graphitization and tailor microstructures. However, post-treatment at elevated temperatures often leads to the formation of metallic residues or carbides that are not fully removable by standard washing protocols, potentially compromising the long-term cycling stability of HCs [180]. Moreover, even when successful removal is achieved, the environmental footprint and additional processing costs associated with the use of metal salts cannot be overlooked. An alternative strategy involves introducing metal salts exclusively during the low-temperature pyrolysis phase (<600 °C) to influence early-stage C formation. Subsequent removal of the salts prior to high-temperature treatment may minimize environmental and structural drawbacks. Though promising, this sequential approach warrants further empirical validation to confirm its feasibility and effectiveness.

#### 5.2.3. Advanced Carbonization Technology

**Figure 26:**
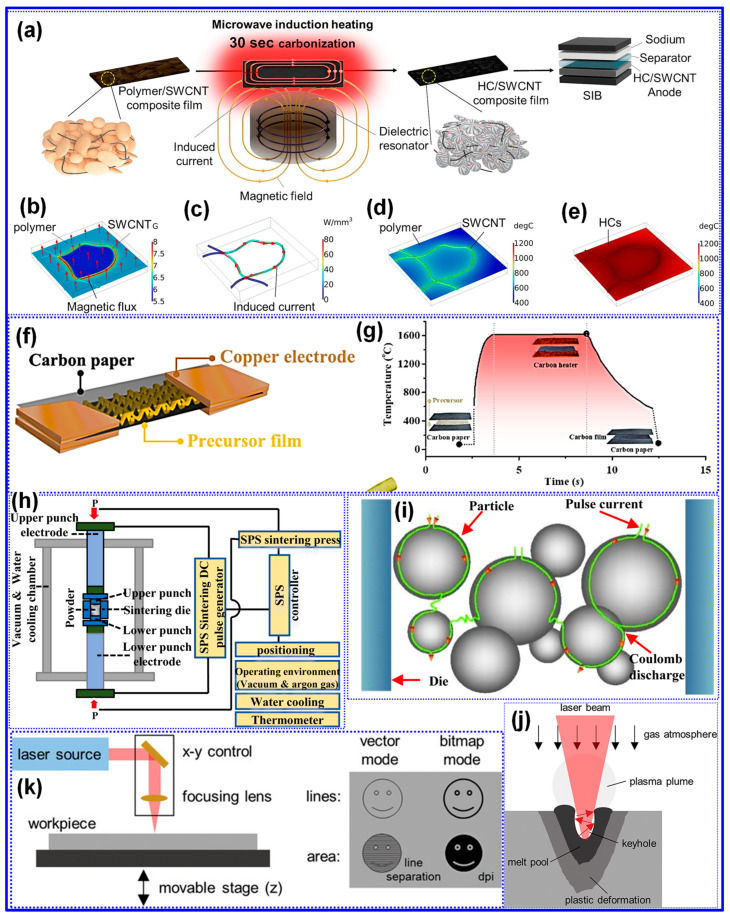
(**a**–**e**) Schematic representation of the HC synthesis for SIBs via microwave induction heating, illustrating the magnetic flux vector, field amplitude, induced current pathways, spatial heating power density, and resulting temperature distribution within the sample. Adapted from [182]. Copyright 2024, Elsevier B.V. (**f**,**g**) Diagram of Joule heating setup with associated time–temperature response curves. Adapted from [186]. Copyright 2024, Elsevier B.V. (**h**,**i**) Diagram of the SPS setup along with the mechanism of annealing. Adapted from [181]. Copyright 2021, PNAS license. (**j**,**k**) Diagram depicting the fundamental working mechanism of the laser system and its interaction dynamics with material surfaces during processing. Adapted from [185]. Copyright 2023, Wiley VCH.

LHCs are typically synthesized via direct thermal treatment of lignin-based precursors at temperatures exceeding 1000 °C for extended periods [101,115]. However, such conventional approaches suffer from low thermal efficiency and considerable energy demands, largely due to their slow ramping rates and prolonged dwell times [181]. In response to these challenges, several high-throughput heating technologies, such as microwave induction [182], joule heating [183], spark plasma sintering [184], and laser-based methods [185], have emerged as promising alternatives for C material fabrication. These advanced methods offer significantly reduced processing times, lower energy consumption, and improved control over microstructural evolution. As shown schematically in Figure 26, many of these techniques are readily applicable to HC synthesis. Notably, Ryoo’s group [182] demonstrated a modified microwave induction process that enabled HC production within 30 sec (Figure 26a–e), attributed to efficient volumetric heating of the precursor mixture. The resulting materials exhibited superior Na storage performance and ICE compared to conventionally synthesized counterparts. Joule heating, likewise, has shown growing potential for energy-efficient and rapid HC fabrication [186,187].

Luo’s group [186] synthesized LHCs by using phenolic resin as a C precursor and applying Joule heating at 1600 °C (Figure 26f,g). In comparison with traditional thermal carbonization, Joule heating enabled rapid processing with markedly reduced energy consumption. The resulting LHCs exhibited notable structural advantages, including lower defect density, diminished O-content, and a higher ratio of sp^2^-hybridized C. Importantly, the Joule-heated samples displayed surface-aligned graphite layers, unlike the disordered graphitic domains typically formed via conventional methods.

In addition to Joule and microwave induction heating, Zhen’s team [181] employed a multifield-regulated spark plasma sintering (SPS) technique to synthesize LHC characterized by low defect density, minimal porosity, and reduced O-content (Figure 26h,i). The SPS process was found to significantly accelerate carbonization kinetics, resulting in LHCs with superior electronic conductivity, elevated graphitization levels, and decreased SSA and O-content. These features translated to a high ICE of 88.9% and excellent rate performance, delivering 136.6 mAh g^−1^ at a current density of 5 A g^−1^. Complementarily, Devi’s team [185] demonstrated the use of laser carbonization for microscale patterning of C, allowing precise fabrication of functional C architectures (Figure 26j,k).

In conclusion, the adoption of advanced carbonization technologies facilitates the rapid fabrication of HCs with tunable microstructures from lignin and other biomass precursors through ultrafast heating regimes. These emerging methods offer distinct advantages, including enhanced energy efficiency, reduced processing times, and increased throughput. Nevertheless, current limitations persist, particularly in terms of scalability, as the fundamental characteristics of these heating techniques restrict their applicability to continuous, large-scale manufacturing. Moreover, the propensity of ultrafast heating to generate highly graphitized C structures may be suboptimal for SIB applications, where a balance between disorder and graphitic order is critical. Fine control over structural parameters also remains a technical hurdle, underscoring the need for further process optimization.

#### 5.2.4. Template-Assisted Tuning

**Figure 27:**
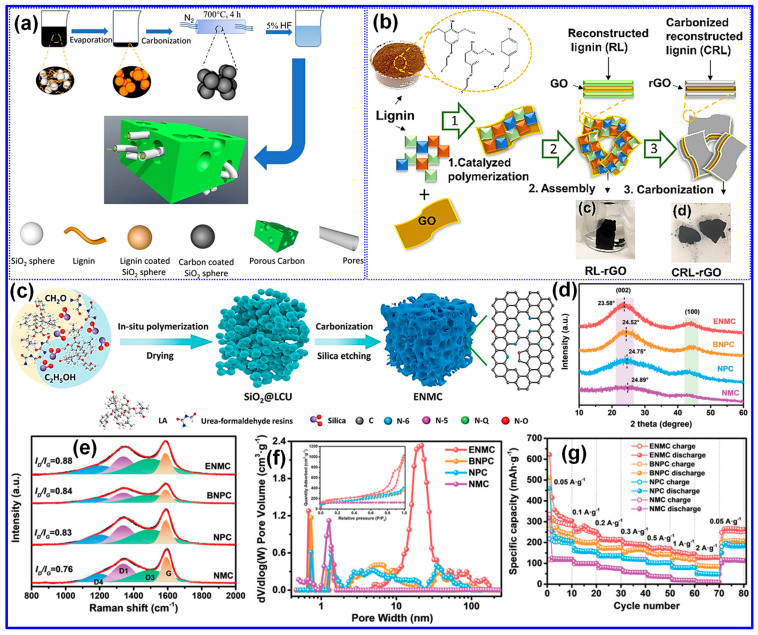
Schematic representations illustrating the synthesis pathways of LHCs by (**a**) SiO_2_. Adapted from [145]. Copyright 2019, IOP publishing. (**b**) GO. Adapted from [192]. Copyright 2021, Springer Nature. (**c**) Polycondensation-based templates. (**d**) XRD, (**e**) Raman, (**f**) pore size distribution, and (**g**) rate capability of LHCs prepared by polycondensation-based template from 0.05–2 A g^−1^. Adapted from [193]. Copyright 2022, Wiley VCH.

Template-assisted strategies for the synthesis of C materials typically involve impregnating or coating a C precursor onto a structural template, followed by carbonization under inert conditions and subsequent removal of the template via thermal degradation or chemical etching [188]. Owing to its superior ability to control morphology and porosity, this method has been widely employed for engineering the structural features of C-based materials [189,190]. For instance, Du and collaborators [145] utilized a SiO_2_ hard-template method to fabricate LHC with a honeycomb-like architecture and uniform nanopores. As illustrated in Figure 27a, lignin was combined with SiO_2_ particles of controlled size, subjected to ultrasonic treatment, and then dried by solvent evaporation. The composite was then pyrolyzed under a N_2_ atmosphere, and the SiO_2_ was subsequently removed using HF etching to yield the final LHCs. The authors demonstrated that both SSA and P_v_ could be effectively tuned by modifying the particle size of the template. The optimized LHC achieved a reversible capacity of 205 mAh g^−1^ at a current density of 0.05 A g^−1^ when tested as an anode for SIBs.

Notably, certain templates also exhibit catalytic properties, thereby influencing the microstructure of the resulting C materials [191,192]. For instance, Jiang’s group [192] employed GO as a dual-functioning template and catalyst to synthesize LHC nanosheets with minimized defects, reduced O-content, and fewer micropores compared to non-templated samples. As illustrated in Figure 27b, during the hydrothermal process, lignin partially depolymerized into smaller fragments, which subsequently interacted with O-containing functional groups on the GO surface. This interaction facilitated repolymerization, and upon carbonization, the GO sheets acted as structural nucleation sites, guiding the formation of a low-defect C framework. The resultant material demonstrated superior electrochemical performance, achieving a reversible capacity of 265 mAh g^−1^ at 0.05 A g^−1^ and retaining 170 mAh g^−1^ at 1 A g^−1^.

Huang’s group [193] proposed a polycondensation template method to finely tailor the graphitic nanodomains and mesoporous structure within amorphous LHCs. The technique entails pyrolysis of a composite composed of SiO_2_ and a lignin–amine–urea–formaldehyde resin system (Figure 27c). Through electrostatic interactions between amine-functionalized lignin and SiO_2_, coupled with in situ polycondensation of lignin with urea and formaldehyde, the resulting LHC exhibits enlarged interlayer spacing (Figure 27d), a higher density of structural defects (Figure 27e), and a well-connected mesoporous framework (Figure 27f).

As expected, these tailored microstructural features endow the resulting LHC with enhanced electrochemical performance, delivering a high reversible capacity of 234 mAh g^−1^ at 0.1 A g^−1^ and remarkable rate performance of 129 mAh g^−1^ at 2 A g^−1^ (Figure 27g). Beyond this study, numerous reports have explored template-based strategies for designing LHCs [189]. However, despite the ability of template methods to modulate microstructure, the electrochemical performance of LHCs synthesized via this route still requires significant enhancement.

In conclusion, it is well-established that regulating the preparation process is a critical and effective strategy for synthesizing LHCs with superior electrochemical performance. Nevertheless, several challenges remain and warrant further investigation in future studies. First, many of the existing process optimization approaches involve the use of additional reagents, complex procedural steps, and increased production costs. Therefore, achieving a practical balance between performance enhancement and economic feasibility is essential, particularly for large-scale applications. Second, to enable the rational design of high-performance LHCs, it is imperative to systematically elucidate the relationships between processing conditions and the resulting microstructures. Finally, future strategies should aim to develop LHCs with well-rounded and comprehensive electrochemical properties, rather than excelling in a single metric, to fully meet the requirements of SIB systems.

### 5.3. Post-Treatment Methodology

Following the synthesis of HCs, their microstructure and electrochemical performance can be further optimized through advanced post-treatment strategies. Although traditional techniques such as washing, grinding, and drying have demonstrated some capacity to alter HC properties [106,121], they are not the central focus of this review. Instead, this section highlights four innovative post-synthesis modification approaches, namely, surface engineering, Joule heating, coating methodologies, and CVD, that offer enhanced control over HC structure and functionality.

#### 5.3.1. Surface Engineering

**Figure 28:**
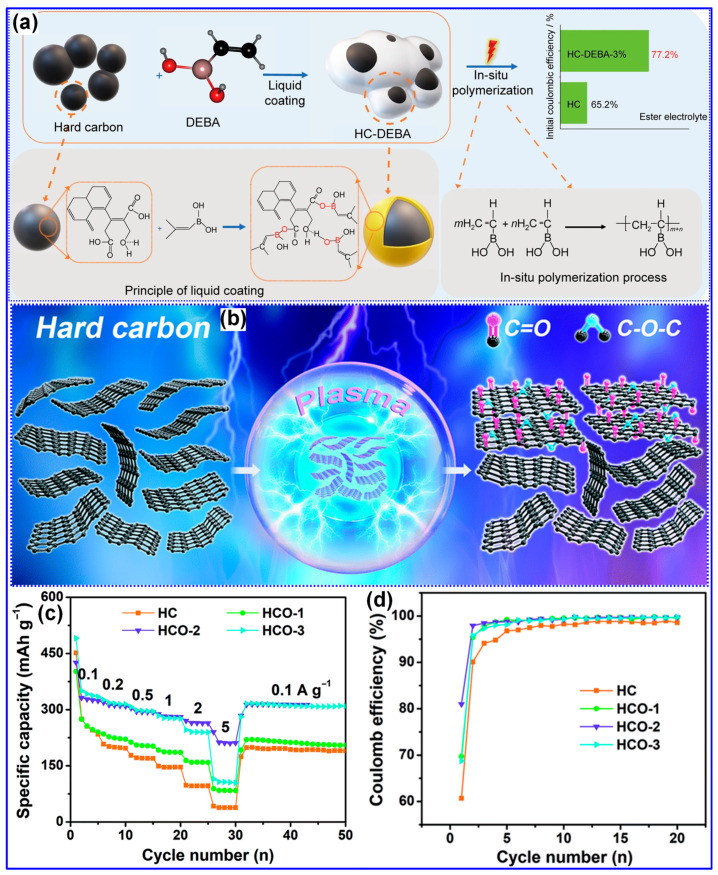
(**a**) Schematic representation of surface engineering on HC via in situ electro-polymerization of 2,2-dimethylvinyl boric acid. Adapted from [195]. Copyright 2022, Springer Nature. (**b**) Schematic illustration of low-temperature O_2_ plasma treatment applied to HC and the resulting structural modifications. (**c**,**d**) Comparative analysis of the electrochemical performance of HCs with and without surface treatment. Adapted from [196]. Copyright 2022, Royal Society of Chemistry.

Surface characteristics of HC, particularly the concentration of O-containing functional groups and the extent of structural defects, exert a significant influence on its electrochemical behavior, including ICE and cycling performance. These surface attributes are closely linked to the formation and composition of the SEI, which, in turn, dictates the interfacial storage dynamics of Na ions. As such, surface engineering has emerged as a powerful strategy for deliberate modification of HC surface characteristics and has attracted increasing research interest [194]. Notably, Yu and co-workers [195] introduced a novel surface engineering method based on in situ electro-polymerization, the schematic of which is depicted in Figure 28a.

Initially, 2,2-dimethylvinyl boric acid (DEBA), bearing both vinyl (C=C) and boronic hydroxyl (B-OH) functional groups, was employed to alter the surface of HC through a liquid-phase coating strategy. During this process, B-OH groups in DEBA covalently bonded with O-functional groups on the HC surface. Upon subsequent electrochemical cycling, electron transfer activated the vinyl groups, generating free radicals that initiated in situ polymerization. The resulting polymeric layer acted as a protective interface, mitigating excessive electrolyte decomposition and promoting the formation of a thinner and more stable SEI. Consequently, the ICE of the HC increased markedly from 65.2 to 77.2%.

**Figure 29:**
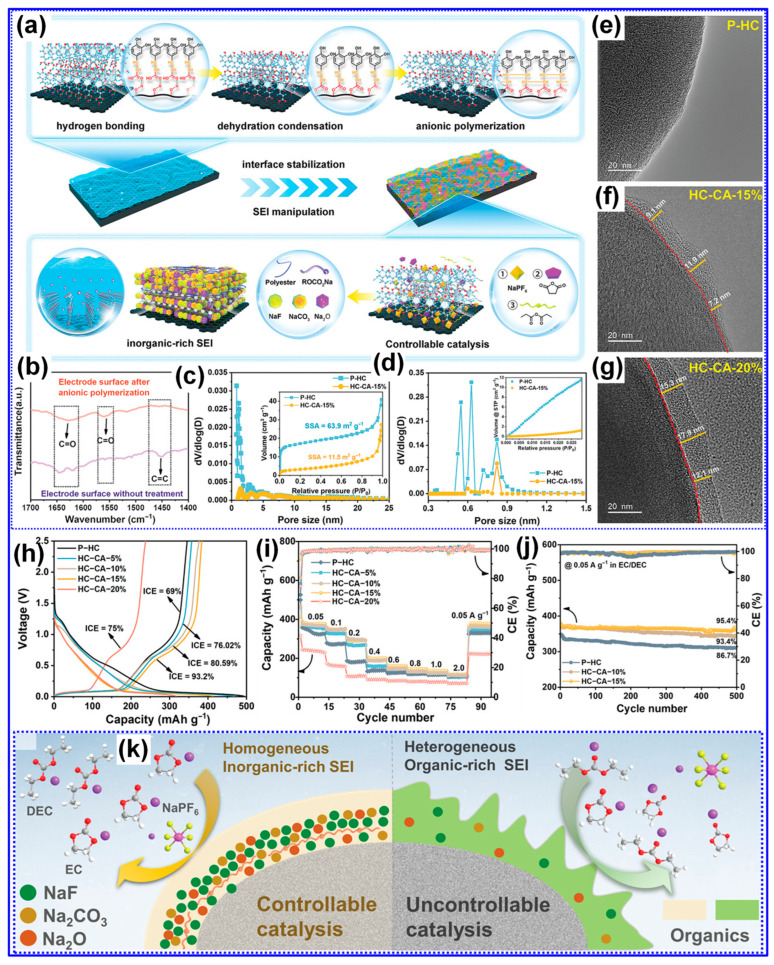
(**a**) Schematic representation of the surface modification strategy involving the grafting of polymerized caffeic acid onto HC. (**b**) FTIR analysis of HCs with or without surface modification. Pore size distribution of HCs from (**c**) N_2_ and (**d**) CO_2_ adsorption/desorption isotherms. (**e**–**g**) TEM micrographs of HCs with and without surface modification. (**h**–**j**) Electrochemical performance comparison of different HC materials. (**k**) Illustration of SEI formation mechanisms of various surfaces. Adapted from [197]. Copyright 2023, Wiley VCH.

Surface oxygenated functional groups on HCs are instrumental in governing the structure and composition of SEI. Leveraging this understanding, Liu and collaborators [197] proposed a novel interfacial catalysis approach aimed at constructing a uniform, multilayered, and inorganic-rich SEI. This was achieved by precisely grafting carbonyl groups onto the HC surface. As depicted in Figure 29a, the in situ introduction of carbonyl functionalities involved a sequence of surface reactions, including hydrogen bonding adsorption, dehydration condensation, and anionic polymerization.

FTIR, pore size distribution analysis, and TEM (Figure 29b–g) confirmed the successful deposition of a polymerized caffeic acid layer enriched with carbonyl functionalities, accompanied by a reduction in SSA and P_v_. The optimized HC sample delivered a high reversible capacity of 379.6 mAh g^−1^, an ICE of 93.2%, and excellent cycling stability, retaining 95.4% of its capacity after 500 cycles at 0.05 A g^−1^ (Figure 29h–j). Further characterization supported the proposed interfacial catalysis mechanism, suggesting that the carbonyl groups act as catalytic “anchor points” to facilitate controlled electrolyte decomposition and direct SEI formation (Figure 29k). In contrast, Shen’s team [196] employed a low-temperature O_2_ plasma (LTOP) treatment (Figure 28b) to introduce carbonyl groups. This approach resulted in a C surface with a higher concentration of carbonyl functionalities and improved structural order with fewer defects. The LTOP-treated HC exhibited a markedly improved ICE of 80.9%, compared to 60.6% for the untreated sample, and achieved a reversible capacity of 331 mAh g^−1^ at 0.1 A g^−1^ (Figure 28c,d).

Beyond the previously discussed strategies, additional surface engineering methods, such as CO_2_-assisted ball milling [198], annealing [199], ozonolysis [200], have been investigated for their potential to improve HC materials. These techniques have collectively demonstrated efficacy in enhancing ICE and cycling performance. However, it is essential to acknowledge the potential drawbacks of surface modifications, including diminished electronic conductivity and reduced availability of active sites, which could impair capacity and rate performance. Moving forward, the development of cost-effective, scalable, and straightforward surface engineering approaches remains a critical objective for practical implementation.

#### 5.3.2. Joule Heating

**Figure 30:**
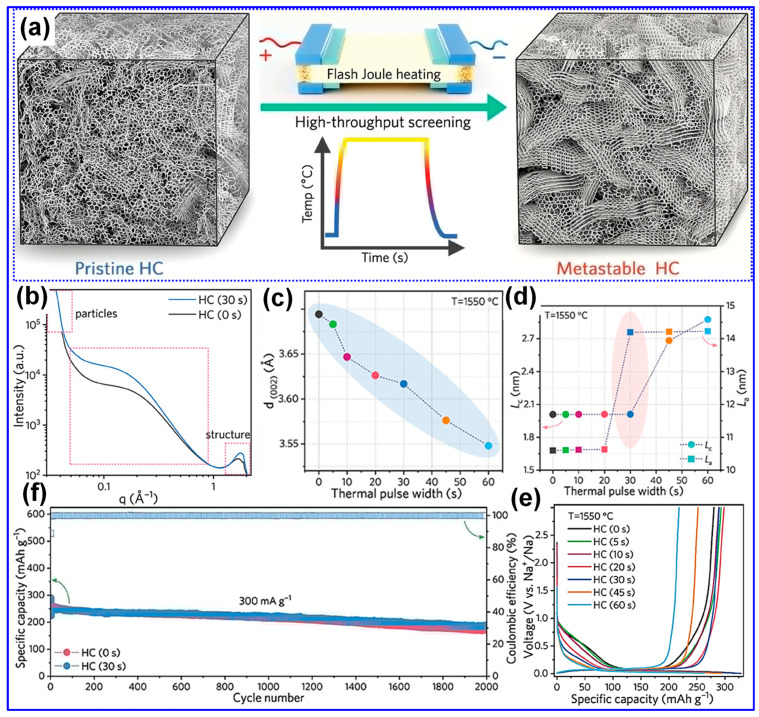
(**a**) Preparation of metastable HC with localized graphitic ordering and closed nanopores using flash Joule heating. Comparison of (**b**) SAXS scattering, (**c**) d_002_, (**d**) L_a_ and L_c_, (**e**) first charge/discharge plots, and (**f**) cycle performance of pristine and modified HCs. Adapted from [205]. Copyright 2024, Wiley VCH.

Joule heating has emerged as a rapid thermal treatment technique garnering attention across diverse applications, including the fabrication of energy storage materials [201] and battery metal recovery [202], among others [203]. This technology has also shown notable efficacy in modifying and reconstructing C materials, owing to its unique localized and ultrafast heating mechanism [134,204]. For instance, Noh and colleagues [204] demonstrated the restoration of sp^2^ hybridization in graphene fibers via joule heating, wherein electric current selectively repaired defect sites by flowing through conductive domains. Building on this, Liu and co-workers [205] developed a flash joule heating method to synthesize metastable HCs with finely tuned microstructures and phase compositions (Figure 30a). By modulating peak temperatures and pulse durations, they achieved controlled graphitization and closed pore formation. SAXS, XRD, and Raman analyses verified the presence of short-range ordered graphitic domains and optimized closed pore structures (Figure 30b–d). Impressively, the resulting HC exhibited a low-voltage plateau capacity of 325 mAh g^−1^, one of the highest reported to date (Figure 30e), along with superior cycling performance (Figure 30f).

**Figure 31:**
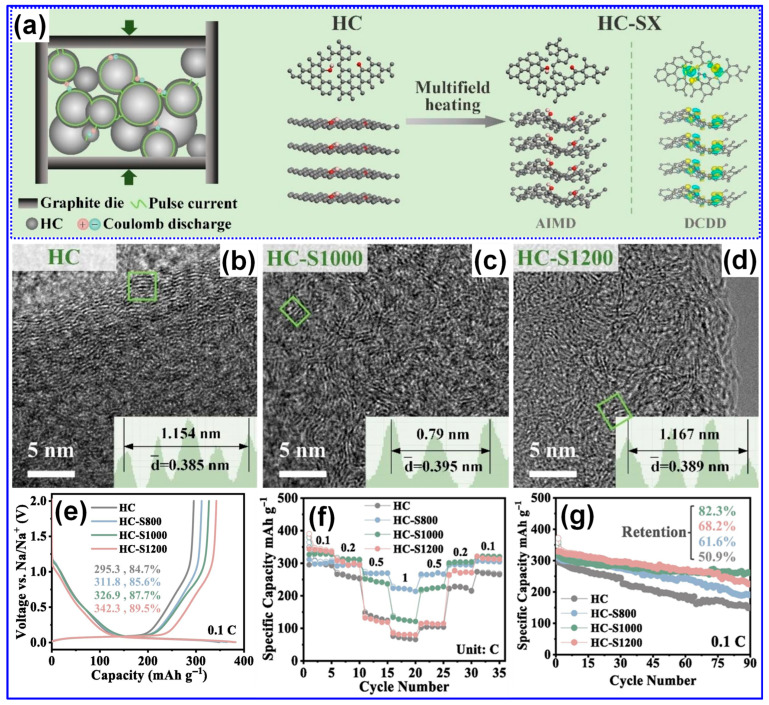
(**a**) Overview of Joule heating post-treatment with multifield sintering, supported by ab initio MD simulations and differential charge density analysis. Comparison of (**b**–**d**) TEM, (**e**) first charge/discharge profiles, (**f**) rate, and (**g**) cycling performances of HCs with and without post-treatment. Adapted from [206]. Copyright 2024, Elsevier B.V.

Song and collaborators [206] proposed an alternative HC microstructure reconstruction technique based on Joule heating. By integrating Joule heating with multifield sintering, they successfully converted thick graphene layers into a disordered, vortex-like architecture comprising curved, thin graphitic domains (Figure 31a). TEM imaging revealed an increase in interlayer spacing post-treatment (Figure 31b–d). MD simulations and charge density analyses suggested that this transformation was driven by intensified interactions between O-containing functional groups and graphene layers under an applied electric field. The optimized HC achieved a high reversible capacity of 342.3 mAh g^−1^, an ICE of 89.5%, and exhibited superior rate performance and cycling activity (Figure 31e–g). These findings underscore the potential of Joule heating as a fast, energy-efficient method for HC tuning, though precise temperature regulation and scale-up remain challenges.

#### 5.3.3. Coating

HCs, in contrast to graphite and soft C, are characterized by a greater density of structural defects, elevated levels of O-containing surface functionalities, and larger SSA. These attributes promote irreversible Na ion adsorption, side reactions, and electrolyte degradation when employed as anodes in SIBs, thereby resulting in diminished ICE and restricted reversible capacity [207]. To mitigate these limitations, various methodologies have been proposed, with surface coating emerging as a particularly promising strategy [208]. By modifying the interface between the HC and electrolyte, this technique minimizes direct contact, delivering two primary advantages: (1) reduced Na loss and suppressed parasitic reactions due to decreased defect sites and SSA and (2) inhibited electrolyte decomposition via the formation of a stable SEI layer [209]. Widely utilized coating materials include inert metal oxides, organic layers, pitch, and asphalt [210,211].

Metal oxides are extensively utilized as surface coatings for both anode and cathode in SIBs to enhance interfacial stability and electrochemical performance [212,213]. Yu’s team [211] employed a liquid-phase coating method to deposit a uniform amorphous Al_2_O_3_ layer onto HC, which facilitated the formation of a stable SEI. This modification led to a significant improvement in the ICE, rising from 64.7 to 81.1%, and an increased reversible capacity of 321.5 mAh g^−1^ at 50 mA g^−1^. In a related study, Lu’s team [214] utilized atomic layer deposition (ALD) to apply an ultrathin Al_2_O_3_ film. This approach effectively reduced surface defects and suppressed electrolyte degradation, resulting in the formation of a uniform and robust SEI. Consequently, the ICE improved from 67 to 75%, and the reversible capacity increased from 260.9 to 355 mAh g^−1^.

**Figure 32:**
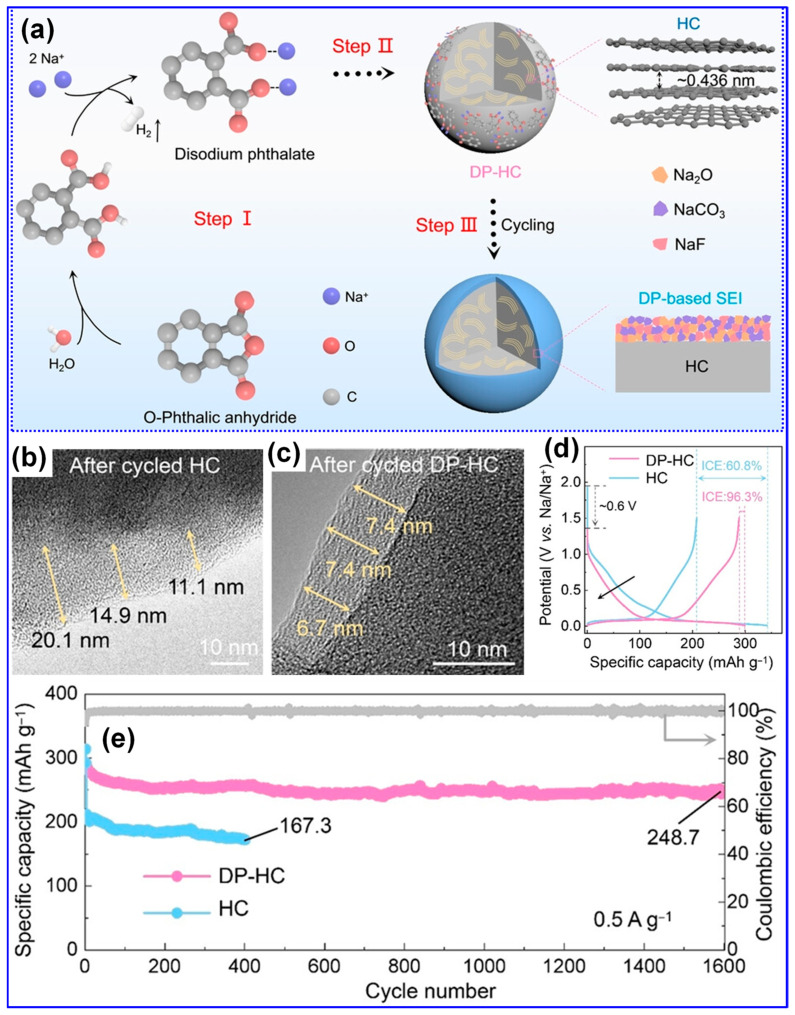
(**a**) Preparation route of disodium phthalate-coated HC and schematic representation of SEI formation. (**b**,**c**) TEM micrographs after cycling, (**d**) first charge/discharge profiles, and (**e**) cycle performance of pristine and coated HCs. Adapted from [209]. Copyright 2024, Wiley VCH.

Although metal oxide coatings on HC can enhance the ICE and reduce irreversible capacity loss, they fall short in addressing Na ion consumption during SEI formation. To address this issue, Sun’s group [209] introduced a disodium phthalate coating that not only compensates for Na loss but also promotes the formation of a uniform and ultrathin SEI (~7.4 nm), as illustrated in Figure 32a–c. This strategy significantly increased ICE from 60.8 to 96.3% and delivered a reversible capacity of 248.7 mAh g^−1^ at 0.5 A g^−1^ (Figure 32d,e). Soft C has also been explored as an alternative coating material for HCs. For instance, Hu’s group [215] used soft C derived from toluene pyrolysis, resulting in a substantial ICE increase from 53 to 83%. Likewise, Li and colleagues [216] applied a coal tar pitch coating to activated C, forming a denser surface layer and improving performance, with ICE rising from 51 to 80% and reversible capacity from 246 to 391 mAh g^−1^.

**Figure 33:**
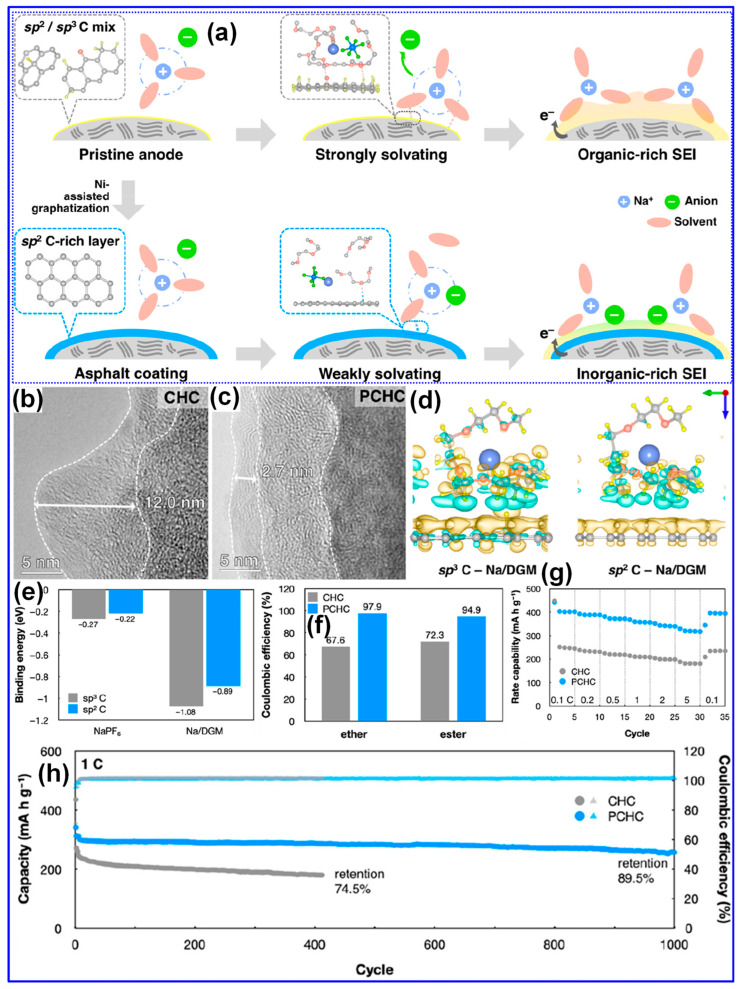
(**a**) Schematic of asphalt-derived sp^2^/sp^3^ C tuning and its impact on SEI formation. (**b**,**c**) TEM micrographs after cycling of pristine and coated HCs. (**d**,**e**) Charge density variation and theoretical BE of sp^2^/sp^3^ C. (**f**) ICE, (**g**) rate and (**h**) cycling performances of pristine and asphalt-coated HCs. Adapted from [210]. Copyright 2024, American Chemical Society.

Zhao and colleagues [210] recently introduced a weakly solvating interfacial engineering approach to modulate the SEI on HC. By employing Ni-assisted carbonization of asphalt, they fabricated a soft C coating characterized by a precisely controlled sp^2^/sp^3^ hybridization ratio and tunable structural disorder (Figure 33a). Compared to the organic-rich SEI typically observed on uncoated HC, the coated surface facilitated the formation of a thinner, more uniform, and predominantly inorganic SEI. This transformation is attributed to modified solvent adsorption energies, which favor the integration of anionic species into the primary solvation sheath, thereby establishing a weakly solvating electrolyte/C interface (Figure 33b–e). Electrochemical evaluations demonstrated a significant enhancement in ICE, achieving 97.9 (vs. 67.6% for pristine HC) in ether electrolytes and 94.9 (vs. 72.3%) in ester electrolytes, in addition to improved rate capability and cycling performance (Figure 33f–h). This work provides a promising coating strategy for HC and expands the practical application of weak solvation design concepts.

Surface coatings, while maintaining the intrinsic structural integrity of HC, effectively mitigate electrolyte decomposition and irreversible Na ion adsorption, thereby enhancing ICE, reversible capacity, and cycling durability. Given these merits, coating strategies present considerable potential for practical implementation. Nonetheless, the development of simpler, more efficient, and economically viable coating technologies capable of producing uniform and precisely controllable surface layers remains an important challenge.

#### 5.3.4. CVD

**Figure 34:**
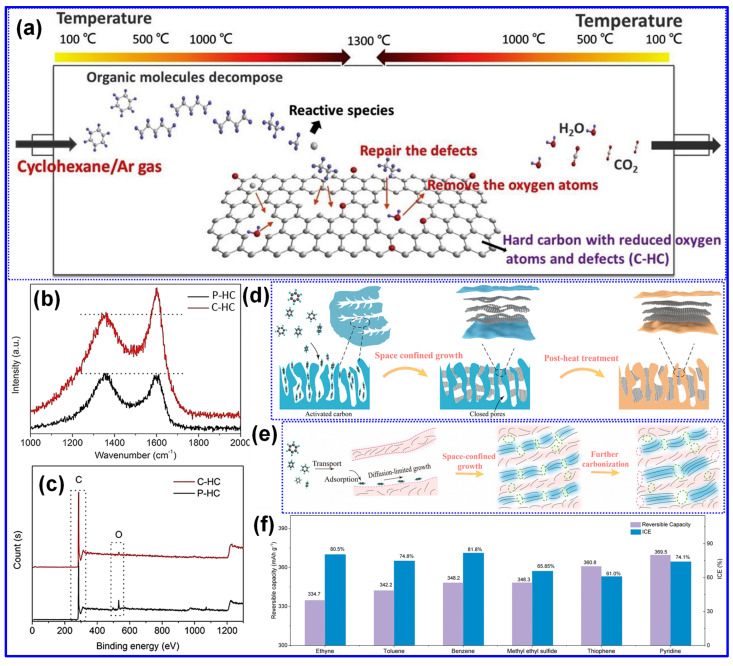
(**a**) Schematic of CVD-based tuning of O-content and defects in HC. (**b**) Raman and (**c**) XPS spectra of HCs before and after CVD. Adapted from [218]. Copyright 2019, Elsevier B.V. (**d**) Schematic of filled HC preparation using the SC-CVD approach. (**e**) Growth mechanism of benzene-derived C under space-confined conditions. (**f**) Summary of reversible capacity and ICE for filled HCs from various C sources. Adapted from [219]. Copyright 2023, Royal Society of Chemistry.

As previously noted, surface defects and O-containing functionalities are primary contributors to irreversible capacity losses and reduced ICE in HC. Beyond conventional coating and surface modification techniques, CVD has demonstrated considerable effectiveness in addressing these structural and chemical limitations [217,218]. Sun et al. [218] utilized a cyclohexane/Ar atmosphere during CVD to decrease surface O-content and heal structural defects (Figure 34a–c). This modification was facilitated by reactive intermediates generated from the thermal decomposition of organic species, resulting in a marked improvement of the ICE to 85% compared to 70% for untreated HC. Similarly, Song and co-workers [217] synthesized a curly, microporous graphene coating on HC through methanol vapor pyrolysis at 1140 °C. This engineered graphene layer enhanced electronic conductivity and effectively repaired surface defects, yielding an ICE of 88.6% and a high-rate capacity of 145.8 mAh g^−1^ at 5 A g^−1^.

Beyond defects, pore structure and graphitic domains significantly influence the electrochemical performance of HC. Chen and collaborators [219] employed a space-confined CVD (SC-CVD) technique to simultaneously form graphitic domains and closed pores within activated C, resulting in a novel “filled HC.” During synthesis, benzene preferentially adsorbs onto micropore walls and, under high temperatures, is catalytically converted into graphene layers, forming closed pores concurrently (Figure 34d). The SC-CVD parameters enable precise tuning of the graphitic structure and pore configuration. The optimized filled HC demonstrated a high reversible capacity of 435.5 mAh g^−1^ and exceptional cycling stability. Mechanistic analysis revealed that benzene pyrolysis under confined conditions follows a mass-transport-controlled growth, yielding large, thin-layered graphitic domains and closed pores (Figure 34e). Additionally, the study evaluated different C sources, nonaromatic organics, benzene/derivatives, and heterocyclic compounds and identified benzene derivatives as the most effective precursors for achieving superior electrochemical performance (Figure 34f).

**Figure 35:**
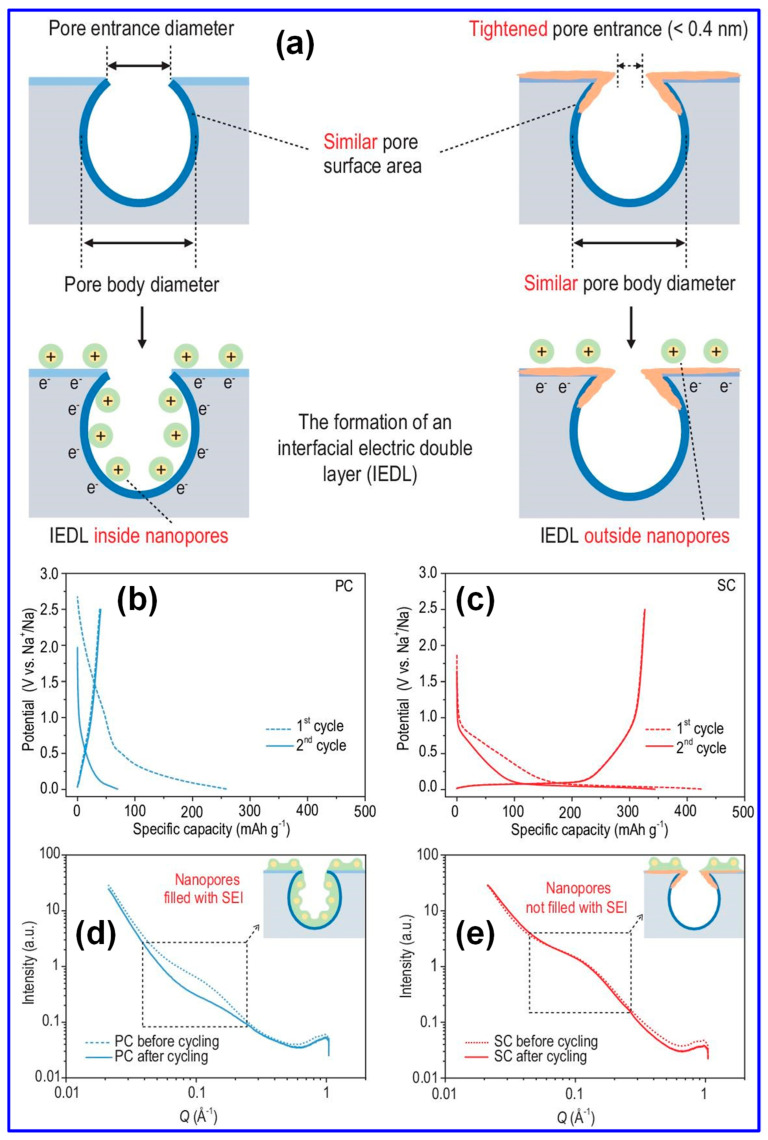
(**a**) Schematic of sieving C preparation and interfacial electric double layer comparison between pristine and sieving HC. Comparison of (**b**,**c**) charge/discharge profiles for the first two cycles and (**d**,**e**) SAXS patterns between pristine and sieving HCs. Adapted from [220]. Copyright 2022, Oxford University Press.

Leveraging the same CVD approach, Li and colleagues [220] devised an innovative “sieving” HC by selectively constricting pore entrances rather than completely filling the pores (Figure 35a). This architectural modification effectively restricts electrolyte infiltration, thereby suppressing SEI formation within the pores. In contrast, Na ions retain unobstructed access to the internal pore structure, promoting Na clustering and enabling a substantial plateau capacity (Figure 35b–e). The sieving HC exhibited an outstanding plateau capacity of 400 mAh g^−1^. As a mature and operationally straightforward post-treatment, CVD holds considerable promise for scalable practical implementation; nonetheless, cost and safety considerations remain pivotal factors for its broader adoption in advanced HC design for SIBs.

Beyond the aforementioned strategies, various other post-treatment methods have been explored to optimize HC, including mechanical processing to adjust pore size, SSA, and defect density [221], as well as secondary carbonization in reducing atmospheres [222]. As the final opportunity to modify HC microstructure before application, post-treatment plays a pivotal role in both research and industrial contexts. For instance, Kuraray Co. (Tokyo, Japan) routinely applies a CVD post-treatment to improve HC properties by repairing defects and reducing SSA. With the advent of emerging technologies, microstructural tuning of HC via post-treatment has become increasingly precise. Nevertheless, future research must focus on achieving greater performance enhancements through cost-effective and simplified post-treatment processes.

## 6. Conclusions and Future Prospects

SIBs have emerged as a promising alternative to LIBs for large-scale energy storage and low-speed electric mobility, primarily owing to their lower cost and the abundance of Na resources. The performance of SIBs, however, critically depends on the development of suitable anode materials. Among the various candidates explored, HCs stand out due to their structural tunability, simple synthesis processes, and favorable Na storage behavior. Despite these advantages, challenges persist, particularly concerning high production costs and less-than-ideal electrochemical performance. Lignin, a naturally occurring phenolic macromolecule abundantly present in lignocellulosic biomass, offers a renewable, C-rich, and low-cost precursor for HC synthesis. Moreover, lignin utilization does not compete with food resources, further enhancing its sustainability and appeal. Consequently, LHCs have attracted increasing attention as potential anodes for SIBs. Optimizing their structure and electrochemical properties is therefore crucial for advancing next-generation SIB technologies. This review begins by discussing the structural characteristics, classification, and pyrolysis behavior of lignin. It then provides an overview of the fundamental features and Na storage mechanisms of HCs to establish a foundation for understanding LHC systems. Subsequently, it systematically summarizes the synthesis routes, structural attributes, and influence of feedstock and processing conditions on LHC performance. Particular focus is given to optimization strategies, including precursor pretreatment, process control, and post-synthesis modifications, to guide performance enhancement. Finally, although considerable progress has been made, large-scale commercialization of LHCs for SIB applications remains challenging. Accordingly, this review concludes with a detailed discussion of future research directions and practical recommendations aligned with current technological trends and industrial requirements.

1. A deeper understanding of the interrelated “property–structure–performance” relationships in LHCs for SIBs remains crucial yet incomplete. Although correlations between feedstock characteristics and the resulting C structures, and between microstructural features and electrochemical behavior, form the conceptual foundation for rational material design, the fundamental mechanisms governing lignin carbonization are still not fully clarified. Previous studies have examined how specific physicochemical parameters, including molecular weight, O-containing functional groups, and ash content, influence the microstructure of LHCs. However, the primary factors dictating microstructural evolution remain ambiguous. Moreover, the exact contribution of distinct structural motifs to Na storage processes has yet to be conclusively established, hindering the systematic optimization of LHC performance. Addressing these uncertainties will require the integration of advanced characterization techniques, in situ analytical tools, and multiscale theoretical modeling to unravel these complex interdependencies and enable the rational design of high-performance, application-ready LHC anodes for next-generation SIBs.
2. Homogenization of lignin should be prioritized as a fundamental objective in pretreatment strategies, given the significant influence of lignin’s intrinsic properties on the structural evolution and electrochemical performance of LHCs. However, lignin’s intrinsic heterogeneity, rooted in both its botanical origin and extraction methodology, complicates the comprehensive elucidation of the “property–structure–performance” paradigm and poses challenges for achieving consistent structural control during LHC synthesis. Accordingly, the advancement of large-scale LHC production is contingent upon the reliable availability of lignin with uniform physicochemical characteristics. While current pretreatment methodologies, encompassing purification, morphology regulation, functional group adjustment, heteroatom doping, and feedstock blending, primarily aim to enhance LHC performance, they seldom address lignin uniformity explicitly. To overcome this limitation, there is an urgent need to develop robust and versatile pretreatment techniques capable of yielding lignin with well-defined and reproducible properties. Notably, an emerging strategy inspired by lignin refinery research involves the depolymerization of lignin into monophenols or polyphenols for subsequent phenolic resin synthesis, potentially facilitating the scalable production of advanced, uniform HCs suitable for practical SIB applications.
3. Feedstock integration represents a promising pathway for the scalable synthesis of LHCs that exhibit a balanced suite of electrochemical properties. For practical implementation, LHCs must combine high specific capacity, extended cycle life, elevated ICE, favorable rate performance, and cost-effectiveness. However, achieving these targets concurrently is inherently challenging, as prevailing optimization techniques, such as pre-oxidation, activation, and surface engineering, tend to improve individual attributes while compromising others. Accordingly, it is imperative to develop approaches that enhance multiple performance parameters in concert. Feedstock integration, which entails the co-processing of lignin with other carbonaceous materials to produce heterogeneous HCs, can deliver synergistic benefits without sacrificing process simplicity or compatibility with existing industrial infrastructure. To fully realize this strategy’s potential, careful co-feedstock selection and comprehensive investigations into the synergistic effects and governing mechanisms are essential.
4. Resolving the inherent trade-offs among high capacity, accelerated Na storage kinetics, and secure plateau potential remains a pivotal challenge in the development of LHCs for SIBs. Typically, the charge–discharge profile of HCs comprises a slope region at potentials above 0.1 V and a plateau region below this threshold. Na storage within the slope region confers rapid ion kinetics and improved operational safety but suffers from diminished ICE. Conversely, the plateau region provides enhanced capacity and ICE yet is hindered by sluggish kinetics and the heightened risk of Na plating. Attaining an optimal balance between these competing attributes is essential for the rational design of high-performance LHCs. Emerging research indicates that expanding the plateau region, improving its Na transport properties, and elevating its operating potential could present viable pathways to resolve this performance paradox. Realizing this potential, however, necessitates a deeper elucidation of the structure–activity relationships governing plateau behavior. Moreover, as energy storage needs increasingly extend to low-temperature environments, where SIBs offer superior performance relative to LIBs, persistent challenges such as dendrite formation and inadequate rate capability must be addressed. Consequently, advancing the low-temperature electrochemical performance of LHCs remains an imperative research priority.
5. While significant progress has been made in developing advanced LHCs for SIBs, practical cost–benefit analysis remains largely neglected. Emerging fabrication methods and microstructure-targeted technologies are often highlighted for their performance advantages, yet their economic and environmental impacts are frequently overlooked. Despite lignin’s renewable nature, LHC production can be energy-intensive and potentially environmentally taxing, calling into question the sustainability claims often associated with these materials. Given that scalability and real-world deployment depend heavily on economic feasibility, the development of LHCs should be accompanied by rigorous cost–benefit evaluations, including output-to-cost and energy-to-price ratios, to ensure practical viability.
6. Machine learning (ML) represents a transformative advance in the rational design and optimization of LHCs for SIB applications. Conventional experimental and analytical methodologies often fall short of fully elucidating the complex “process–structure–performance” interrelationships that arise due to the inherent variability of lignin feedstocks, diverse carbonization pathways, and varying electrochemical testing protocols. By efficiently processing extensive multivariate datasets, ML facilitates the identification of nuanced correlations, thereby supporting systematic material design. When integrated with computational chemistry approaches, such as DFT and MD, ML enhances predictive reliability and expedites simulation workflows. This integration enables high-throughput exploration of pretreatment strategies, dopant incorporation, and thermal treatment parameters. Moreover, ML’s capacity for multi-objective optimization allows simultaneous consideration of critical performance indicators (e.g., specific capacity, ICE, and cycle stability) alongside cost-effectiveness and scalability. Coupled with high-throughput experimentation and in situ characterization, ML empowers deeper mechanistic understanding and accelerates iterative development, significantly advancing the practical realization of LHCs for next-generation SIB systems.

As demonstrated by the studies summarized herein, notable progress has been achieved in advancing LHCs with favorable Na storage characteristics. Nevertheless, several fundamental challenges persist, necessitating sustained research efforts and iterative experimental refinement to enable the practical deployment of LHCs in SIBs. Despite these enduring barriers, LHCs represent a compelling avenue for the continued evolution of SIB technologies. It is hoped that this review will serve as both a conceptual framework for the rational design of next-generation LHCs and an impetus for further exploration of lignin valorization within the broader energy storage research community.

## Figures and Tables

**Table 1:** Comparative analysis of various feedstocks for SIBs [2,25].

Feedstocks		Cost ($/t)	C Yield (%)	Modifiability	Availability	Performance of Derived HCs
Pitch		300	>50	Difficult	Abundant	Poor
Anthracite		50–200	∼90			
Lignocellulose biomass	Cellulose	1000	<10	Normal		Good
	Lignin	300	>40	Easy		
	Starch	500	<10	Normal	_ ^1^	
	Sucrose	400			_ ^1^	
	Coconut shell	650 ^2^	∼25		Insufficient	
Phenolic resin		2000	>40	Easy	Abundant	Excellent

^1^ According to regional regulations, it is prohibited to use this material as an industrial feedstock for producing HCs. ^2^ The cost of coconut shell following low-temperature carbonization (yielding a fixed C content of 70%).

**Table 2:** A detailed comparative analysis of various lignin types [36,38,54].

Classifications	Annual Production (t)	Ash Contents (%)	MW (g mol^−1^)	Polydispersity	Solubility
Lignosulfonate	>1,000,000	4–8	1000–61,000	6–8	Water of wide pH
Organosolv lignin	Pilot scale	<1.5	500–5000	1.5–4.4	Organic solvents, Alkali solutions
Klason lignin	Lab scale	1–3	2000–7000	1.5–7.2	Alkali solutions
Kraft lignin	>55,000,000		1000–5000	2.5–3.5	Alkali solutions, polar organic solvents
Soda lignin	>6000	<2.8	0.8–3000		Alkali solutions
Steam explosion lignin	Lab scale	<4	1000–15,000	2.5–7	
Enzymatic lignin	Pilot scale	1–3	2000–9500	1.5–3.2	

**Table 3:** Factors influencing the structural characteristics and electrochemical performance of LHCs for SIBs.

Variable Factors	Horizontal Factors	Carbonization Conditions	Sodium Storage Performances			Structural Information			Ref.
			Capacity (mAh g^−1^) & Current Density (C_d_, A g^−1^)	ICE (%)	Cycling Stability	Graphitic Domains	Defects	Pv (cm^3^ g^−1^) & S_BET_ (m^2^ g^−1^)	
Lignin types (Different molecular weights (MWs)	Low	Heating rate: 5 °C min^−1^; Annealing time: 2 h; Carbonization temperature: 700 °C	-	-	-	d_002_ = 3.96; L_a_ = 1.20 nm	-	S_BET_ = 6.1	[104]
	Middle					d_002_ = 4.04; L_a_ = 1.24 nm		S_BET_ = 12.3	
	High					d_002_ = 3.91; L_a_ = 1.25 nm		S_BET_ = 0.71	
Lignin types (Different extraction methods)	Kraft lignin (LK)	Heating rate: 5 °C min^−1^; Annealing time: 1 h	C = 181 C_d_ = 0.025	66.4	-	d_002_ = 3.842	I_d_/I_g_ = 2.4	*^b^* P_v_ = 0.04; S_BET_ = 1.6	[89]
	Lignin sulphonates (LS)	Carbonization temperature: 1200 °C	C = 205 C_d_ = 0.025	64.3	-	d_002_ = 3.827		*^b^* P_v_ = 0.16; S_BET_ = 180	
	Lignin sulphonates (LSW)	Heating rate: 5 °C min^−1^; Annealing time: 1 h; *^c^* Carbonization temperature: 600 and 1200 °C	C = 284 C_d_ = 0.025	79.1	Capacity retention is 95.1% after 50 cycles at 0.1 C	d_002_ = 3.797		*^b^* P_v_ = 0.24; S_BET_ = 5.6	
Lignin types (Different molecular weights, oxygenated functional groups, and polarities)	Low MW, high polarity and high carboxyl, phenolic hydroxyl contents	Heating rate: 5 °C min^−1^; Annealing time: 6 h; Carbonization temperature: 1300 °C	C = 268; C_d_ = 0.05	59.5	Capacity retention is 82.9% after 100 cycles at 0.1 A g^−1^	d_002_ = 0.399 nm; L_a_ = 4.57 nm; L_c_ = 0.85 nm	I_d_/I_g_ = 3.49	*^a^* P_v_ = 0.0932 S_BET_ = 117.17	[105]
	Middle MW, middle polarity and high alcohol hydroxyl contents	-	C = 314; C_d_ = 0.05	66.6	Capacity retention is 78.2% after 100 cycles at 0.1 A g^−1^	d_002_ = 0.406 nm; L_a_ = 4.74 nm; L_c_ = 0.88 nm	I_d_/I_g_ = 3.26	*^a^* P_v_ = 0.158 S_BET_ = 185.42	
	High MW, low polarity and low oxygen contents	-	C = 312; C_d_ = 0.05	66.2	Capacity retention is 89.4% after 100 cycles at 0.1 A g^−1^	d_002_ = 0.390 nm; L_a_ = 4.27 nm; L_c_ = 0.95 nm	I_d_/I_g_ = 3.29	*^a^* Pv = 0.1186 S_BET_ = 182	
Lignin types (Different ash contents)	6.4 wt%	Heating rate:5 °C min^−1^; Annealing time: 6 h; Carbonization temperature: 1300 °C	-	45	-	d_002_ = 0.377; L_a_ = 3.65; L_c_ = 0.84	I_d_/I_g_ = 1.75	P_v_ = 0.167; S_BET_ = 278.4	[106]
	1.1 wt%		C = 317; C_d_ = 0.05	87	Capacity retention is 89.3% after 70 cycles at 0.05 A g^−1^	d_002_ = 0.379; L_a_ = 4.47; L_c_ = 1.07	I_d_/I_g_ = 1.56	P_v_ = 0.081; S_BET_ = 117.6	
	*^f^* 1.1 wt%		-	63	-	d_002_ = 0.370; L_a_ = 3.81; L_c_ = 0.93	I_d_/I_g_ = 1.64	P_v_ = 0.027; S_BET_ = 36.5	
	3.9 wt%		-	45	-	d_002_ = 0.383; L_a_ = 3.77; L_c_ = 0.94	I_d_/I_g_ = 1.75	P_v_ = 0.110; S_BET_ = 175.3	
	1.7 wt%		-	42	-	d_002_ = 0.377; L_a_ = 3.61; L_c_ = 0.92	I_d_/I_g_ = 1.79	P_v_ = 0.103; S_BET_ = 165	
*^e^* Morphologies	nano-spherical particles (LS)	Heating rate:5 °C min^−1^; Annealing time: 2 h; Carbonization temperature: 800 °C	C = 281.9; C_d_ = 0.1	50.34	Capacity retention is 49% after 100 cycles at 0.1 A g^−1^	d_002_ = 0.36;	I_d_/I_g_ = 0.7	S_BET_ = 40	[107]
	Carbon nanospheres (CNS)		C = 120.8; C_d_ = 0.1	11.51	Capacity retention is 68.6% after 100 cycles at 0.1 A g^−1^	d_002_ = 0.377;	I_d_/I_g_ = 0.67	S_BET_ = 229	
	Porous Carbon (LCS)		C = 162.1; C_d_ = 0.1	26.63	Capacity retention is 80.8% after 100 cycles at 0.1 A g^−1^	d_002_ = 0.387;	I_d_/I_g_ = 0.83	S_BET_ = 4937	
	Carbon particles (LC)		C = 69.3; C_d_ = 0.1	32.34	Capacity retention is 61.8% after 100 cycles at 0.1 A g^−1^	-	I_d_/I_g_ = 0.71	-	
Carbonization temperatures (Unknown lignin type)	1000 °C	Heating rate: 5 °C min^−1^; Annealing time: 6 h	C = 202.61; C_d_ = 0.05	61	Capacity retention >99% after 700 cycles at 0.3 A g^−1^	d_002_ = 0.383 nm; L_a_ = 3.95 nm; L_c_ = 0.98 nm	I_d_/I_g_ = 1.87	P_v_ = 0.228; S_BET_ = 124.9	[42]
	1100 °C		C = 230.29; C_d_ = 0.05	63	-	d_002_ = 0.382 nm; L_a_ = 4.08 nm; L_c_ = 0.99 nm	I_d_/I_g_ = 1.51	P_v_ = 0.187; S_BET_ = 92.1	
	1200 °C		C = 255.56; C_d_ = 0.05	65	-	d_002_ = 0.376 nm; L_a_ = 4.13 nm; L_c_ = 1.05 nm	I_d_/I_g_ = 0.85	P_v_ = 0.113; S_BET_ = 54.9	
	1300 °C		C = 260.55; C_d_ = 0.05	69	-	d_002_ = 0.375 nm; L_a_ = 4.17 nm; L_c_ = 1.13 nm	I_d_/I_g_ = 0.72	P_v_ = 0.112; S_BET_ = 48.3	
	1400 °C		C = 246.36; C_d_ = 0.05	71	-	d_002_ = 0.374 nm; L_a_ = 4.20 nm; L_c_ = 1.14 nm	I_d_/I_g_ = 0.56	P_v_ = 0.107; S_BET_ = 29.5	
	1500 °C		C = 216.61; C_d_ = 0.05	72	-	d_002_ = 0.365 nm; L_a_ =4.22 nm; L_c_ =1.21 nm	I_d_/I_g_ = 0.47	P_v_ = 0.088; S_BET_ = 14.7	
Carbonization temperatures (Milled wood lignin)	1000 °C	Heating rate: 3 °C min^−1^; Annealing time: 2 h	C = 216.4; C_d_ = 0.02	72.3	-	d_002_ = 0.41 nm; L_a_ = 3.25 nm; L_c_ = 0.96 nm	I_d_/I_g_ = 4.1	S_BET_ = 11.2	[58]
	1200 °C		C = 281.6; C_d_ = 0.02	72.8	-	d_002_ = 0.396 nm; L_a_ = 4.267 nm; L_c_ = 1.01 nm	I_d_/I_g_ = 3.7	S_BET_ = 19.7	
	1400 °C		C = 292.6; C_d_ = 0.02	80.1	-	d_002_ = 0.392 nm; L_a_ = 4.34 nm; L_c_ = 1.16 nm	I_d_/I_g_ = 2.56	S_BET_ = 10.5	
	1600 °C		C = 290.7; C_d_ = 0.02	79.5	-	d_002_ = 0.346 nm; L_a_ = 4.37 nm; L_c_ = 1.62 nm	I_d_/I_g_ = 2.38	S_BET_ = 10.3	
Carbonization temperatures (Softwood kraft lignin)	800 °C	Step 1: Heating rate:3 °C min^−1^; Annealing time: 5 min; Carbonization temperature: 600 °C Step 2: Heating rate:5 °C min^−1^; Annealing time: 20 min; Carbonization temperature: 800~1700 °C	C = 150; C_d_ = 0.03	59	-	d_002_ = 0.401 nm; L_a_ = 1.0 nm	-	S_BET_ = 355	[108]
	1000 °C		C = 260; C_d_ = 0.03	70	-	d_002_ = 0.399 nm; L_a_ = 41.0 nm	-	S_BET_ = 387	
	1200 °C		C = 310; C_d_ = 0.03	89	-	d_002_ = 0.398 nm; L_a_ = 1.1 nm	-	S_BET_ = 94	
	1700 °C		C = 280; C_d_ = 0.03	92	-	d_002_ = 0.367 nm; L_a_ = 1.4 nm	-	S_BET_ = 47	
Carbonization temperatures (Dealkalized lignin)	1000 °C	Heating rate:5 °C min^−1^; Annealing time: 2 h	C = 231.7; C_d_ = 0.05	51.4	As the temperature increases, the cycling stability increases synchronously	d_002_ = 0.386 nm; L_a_ = 1.74 nm	I_d_/I_g_ = 1.91	S_BET_ = 87.2	[60]
	1200 °C		C = 279.8; C_d_ = 0.05	56.3		d_002_ = 0.383 nm; L_a_ = 2.16 nm	I_d_/I_g_ = 1.71	S_BET_ = 56.8	
	1400 °C		C = 284.7; C_d_ = 0.05	54.1		d_002_ = 0.375 nm; L_a_ = 2.27 nm	I_d_/I_g_ = 1.58	S_BET_ = 33	
	1600 °C		C = 307.2; C_d_ = 0.05	68.2		d_002_ = 0.365 nm; L_a_ = 2.57 nm	I_d_/I_g_ = 1.49	S_BET_ = 35.9	
	1800 °C		C = 215.1; C_d_ = 0.05	60.8		d_002_ = 0.346 nm; L_a_ = 3.75 nm	I_d_/I_g_ = 1.41	S_BET_ = 45	
Water content in the atmosphere (Kraft soft wood lignin)	0 g m^−3^	Heating rate:10 °C min^−1^; Annealing time: 1 h; Carbonization temperature: 1000 °C	-	-	-	L_a_ = 2.12 nm	-	P_v_ = 0.051; S_BET_ = 31.76	[109]
	18 g m^−3^							P_v_ = 0.14; S_BET_ = 54.2	
	45 g m^−3^							P_v_ = 0.116; S_BET_ = 78.96	
	75 g m^−3^					L_a_ = 2.13 nm		P_v_ = 0.658; S_BET_ = 112	
	105 g m^−3^					L_a_ = 2.12 nm		P_v_ = 0.082; S_BET_ = 105	
Carbonization atmosphere	N_2_	Heating rate:13 °C min^−1^; Annealing time: 6 h; Carbonization temperature: 1000 °C	C = 260; C_d_ = 0.1 C	81.8	-	d_002_ = 0.40 nm; L_a_ = 2.9 nm; L_c_ = 0.8 nm	*^d^* A_d_/A_g_ = 3.7	S_BET_ = 185	[110]
	Ar		C = 252; C_d_ = 0.1C	80.5		d_002_ = 0.40 nm; L_a_ = 2.9 nm; L_c_ = 0.8 nm		S_BET_ = 21	
	Ar + 5% H_2_		C = 128; C_d_ = 0.1 C	47.4		d_002_ = 0.40 nm; L_a_ = 3.0 nm; L_c_ = 0.8 nm	*^d^* A_d_/A_g_ = 3.6	S_BET_ = 252	
Heating rates (Camphor wood residues)	5 °C min^−1^	Annealing time: 2 h; Carbonization temperature: 1300 °C	C = 242.6; C_d_ = 0.02	60.8	Capacity retention is 84.7% after 50 cycles at 0.02 A g^−1^	d_002_ = 0.363 nm; L_a_ = 3.54 nm; L_c_ = 1.71 nm	I_d_/I_g_ = 1.63	P_v_ = 0.068; S_BET_ = 111.4	[111]
	2 °C min^−1^		C = 270.2; C_d_ = 0.02	67.5	Capacity retention is 96.6% after 50 cycles at 0.02 A g^−1^	d_002_ = 0.366 nm; L_a_ = 3.51 nm; L_c_ = 1.70 nm	I_d_/I_g_ = 1.56	P_v_ = 0.065; S_BET_ = 107.2	
	1 °C min^−1^		C = 289.4; C_d_ = 0.02	76.4	Capacity retention is 95.4% after 50 cycles at 0.02 A g^−1^	d_002_ = 0.371 nm; L_a_ = 3.47 nm; L_c_ = 1.68 nm	I_d_/I_g_ = 1.55	P_v_ = 0.017; S_BET_ = 6.68	
	0.5 °C min^−1^		C = 308.9; C_d_ = 0.02	80.4	Capacity retention is 96.1% after 50 cycles at 0.02 A g^−1^	d_002_ = 0.374 nm; L_a_ = 3.45 nm; L_c_ = 1.67 nm	I_d_/I_g_ = 1.5	P_v_ = 0.018; S_BET_ = 6.33	
	0.25 °C min^−1^		C = 324.6; C_d_ = 0.02	82.8	Capacity retention is 98.4% after 50 cycles at 0.02 A g^−1^	d_002_ = 0.379 nm; L_a_ = 3.4 nm; L_c_ = 1.64 nm	I_d_/I_g_ = 1.48	P_v_ = 0.015; S_BET_ = 3.74	
None	Sodium lignin sulfonate	Step 1: Annealing time: 3 h; Carbonization temperature: 500 °C Step 2: Annealing time: 2 h; Carbonization temperature: 1300 °C	C = 339; C_d_ = 0.1 C	88.3	Capacity retention is 93% after 100 cycles at 0.1 C	d_002_ = 0.398	I_d_/I_g_ = 1.07	S_BET_ = 11.89	[112]
	Lignin	Annealing time: 2 h; Carbonization temperature: 1200 °C	C = 203; C_d_ = 0.025	52.7	Capacity retention is 57.4% after 300 cycles at 0.1 A g^−1^	d_002_ = 0.38; L_a_ = 3.804; L_c_ = 0.592	I_d_/I_g_ = 0.46	S_BET_ = 531.1	[113]
	Byproduct of fuel alcohol production derived from corn stalks	Step 1: Annealing time: 1 h; Carbonization temperature: 400 °C Step 2: Annealing time: 3 h; Carbonization temperature: 1300 °C	C = 338.5; C_d_ = 0.025	76.7	Capacity retention is 83.3% after 100 cycles at 0.05 A g^−1^	d_002_ = 0.375; L_a_ = 2.82; L_c_ = 0.67	I_d_/I_g_ = 1.077	S_BET_ = 6.78	[114]
	Concentrated sulfuric acid hydrolysis lignin from oak wood	Heating rate: 10 °C min^−1^; Annealing time: 6 h; Carbonization temperature: 1300 °C	C = 297; C_d_ = 0.05	68%	Capacity retention is 99.8% after 500 cycles at 2.5 A g^−1^	d_002_ = 0.403 nm; L_a_ = 3.44 nm; L_c_ = 0.9 nm	*^d^* A_d3_/A_g_ = 0.8	P_v_ = 0.04; S_BET_ = 249.8	[115]

*^a^* Closed pore volume (P_v_) values obtained through SAXS measurements. *^b^* P_v_ measured via CO_2_ adsorption–desorption isotherms. *^c^* Lignin was initially thermally annealed at 600 °C for 1 h. Following water washing, the sample underwent a second thermal annealing at 1200 °C for an additional hour. *^d^* The integrated intensity ratio of D band to G band. *^e^* Morphological variations are often accompanied by simultaneous changes in feedstock composition and processing conditions. *^f^* The feedstock for HC1300-pA was prepared via washing with a 0.5 wt% aqueous KOH solution.

## Data Availability

No new data were created or analyzed in this study.
